# Neglected role of hydrogen sulfide in sulfur mustard poisoning: Keap1 S-sulfhydration and subsequent Nrf2 pathway activation

**DOI:** 10.1038/s41598-017-09648-6

**Published:** 2017-08-25

**Authors:** Wenqi Meng, Zhipeng Pei, Yongwei Feng, Jie Zhao, Yongchun Chen, Wenwen Shi, Qingqiang Xu, Fengwu Lin, Mingxue Sun, Kai Xiao

**Affiliations:** 1 Lab of Toxicology & Pharmacology, Faculty of Tropical Medicine and Public Health, Second Military Medical University, Shanghai, 200433 China; 2 China-Japan Union Hospital of Jilin University, Changchun, 130033 China

## Abstract

Sulfur mustard (SM) is a chemical warfare agent and a terrorism choice that targets various organs and tissues, especially lung tissues. Its toxic effects are tightly associated with oxidative stress. The signaling molecule hydrogen sulfide (H_2_S) protects the lungs against oxidative stress and activates the NF-E2 p45-related factor 2 (Nrf2) pathway. Here, we sought to establish whether endogenous H_2_S plays a role in SM induced lesion in mouse lungs and lung cells and whether endogenous H_2_S plays the role through Nrf2 pathway to protect against SM-induced oxidative damage. Furthermore, we also explored whether activation of Nrf2 by H_2_S involves sulfhydration of Kelch-like ECH-associated protein-1 (Keap1). Using a mouse model of SM-induced lung injury, we demonstrated that SM-induced attenuation of the sulfide concentration was prevented by NaHS. Concomitantly, NaHS attenuates SM-induced oxidative stress. We also found that H_2_S enhanced Nrf2 nuclear translocation, and stimulated expression of Nrf2-targeted downstream protein and mRNA levels. Incubation of the lung cells with NaHS decreased SM-induced ROS production. Furthermore, we also found that H_2_S S-sulfhydrated Keap1, which induced Nrf2 dissociation from Keap1, and enhanced Nrf2 nuclear translocation. Our data indicate that H_2_S is a critical, however, being long neglected signal molecule in SM-induced lung injury.

## Introduction

As a major chemical warfare agent since its introduction on the battlefield, sulfur mustard (2,2′-dichlorodiethyl sulfide, SM) has been applied in a series of conflicts in the past 100 years and has produced a large number of casualties and serious medical problems, e.g., Italy invasion of Abyssinia, Japan invasion of China, and Iran-Iraq war^1^. SM was produced and stockpiled by many countries and is likely still the most widely equipped chemical warfare agent in the world^2^. SM is also one of the major components in abandoned chemical weapons (ACW) by Japan in China 70 years ago and imposes a great threat to public health and the environment^3^. Given its ready availability, ease of production and storage, and persistence and stability, SM is also regarded as the most possible agent for use in potential terrorist attacks^4^. Recently SM was used by the Islamic State Jihadist group ISIS against Syrian civilians^5^. Although medical research for prevention and treatment against SM has been continuously performed, no specific antidote currently exists for SM exposure to date.

Exposure to SM causes blistering of the skin and damage to the eyes and lungs^6^. A major target of SM is the lung, and most mortality following SM poisoning is due to pulmonary injuries and associated infections. Both acute (e.g., chest tightness, hacking cough, and rhinorrhea) and delayed (e.g., chronic bronchitis, airway hyperreactvitiy, lung fibrosis and bronchopneumonia) effects have been reported after human exposure to SM^7^. Numerous studies have demonstrated that SM or its analogs induce the process of oxidative damage in the lung tissue of exposed rats^8, 9^. Although the cellular and molecular mechanisms of SM toxicity have not been fully recognized, oxidative stress is considered as the initial and vital process for the damage^10^. Antioxidant therapy has been suggested as one of the most important treatment measures for SM-induced injuries^11^. A variety of thiol/sulfur compounds are being investigated as potential therapeutics of SM poisoning, including *L*-cysteine, thiosulfate, acetylcysteine, and glutathione^11^. As a thiol/sulfur compound, hydrogen sulfide (H_2_S) exhibits well-characterized for antioxidant effects, which have been extensively investigated and are regarded as the principal mechanism underlying its beneficial effects^12^. Thus, we hypothesize that H_2_S may play an important role in SM-induced injuries.

Following nitric oxide and carbon monoxide, hydrogen sulfide is the third gaseous mediator, and has been recognized as a crucial signaling molecule with a wide range of physiological functions affecting most organs in animals and human beings^13^. Hydrogen sulfide is endogenously produced in mammalian tissues mainly by cystathionine β-synthase (CBS) and cystathionine γ-lyase (CSE) from L-cysteine, homocysteine and cystathione. Hydrogen sulfide is also produced by the catalysis of 3-mercaptopyruvate sulfurtransferase (3-MST) with L-cysteine and/or homocysteine^13^. Emerging data suggest that H_2_S improves pulmonary fibrosis^14^, lung ischemia-reperfusion injury^15^, and acute lung injury^16^. According to the literature, these protective effects could be attributed to the following mechanisms: scanvenging the reactive oxygen species (ROS) and reactive nitrogen species (RNS), increasing glutathione, and promoting the translocation of the nuclear transcription factor Nrf2 to induce the activation of numerous detoxifying genes^17, 18^.

Herein, we reported our findings concerning the role of H_2_S in SM-induced injuries. First, the effects of SM on endogenous H_2_S in mouse lungs and lung cells were analyzed. Next, we examined whether H_2_S restored SM-induced injuries through inhibiting oxidative stress. Further, we established whether endogenous H_2_S requires Nrf2 to protect against SM-induced lung oxidative damage. Finally, we explored whether activation of Nrf2 by H_2_S involves antagonism of Keap1. To the best of our knowledge this is the first study that investigates the possible mechanisms of H_2_S and/or the Nrf2 pathway on the experimental validation of SM-induced pulmonary injury.

## Results

### Effect of SM on endogenous the sulfide concentration in mouse lungs

**Figure 1:**
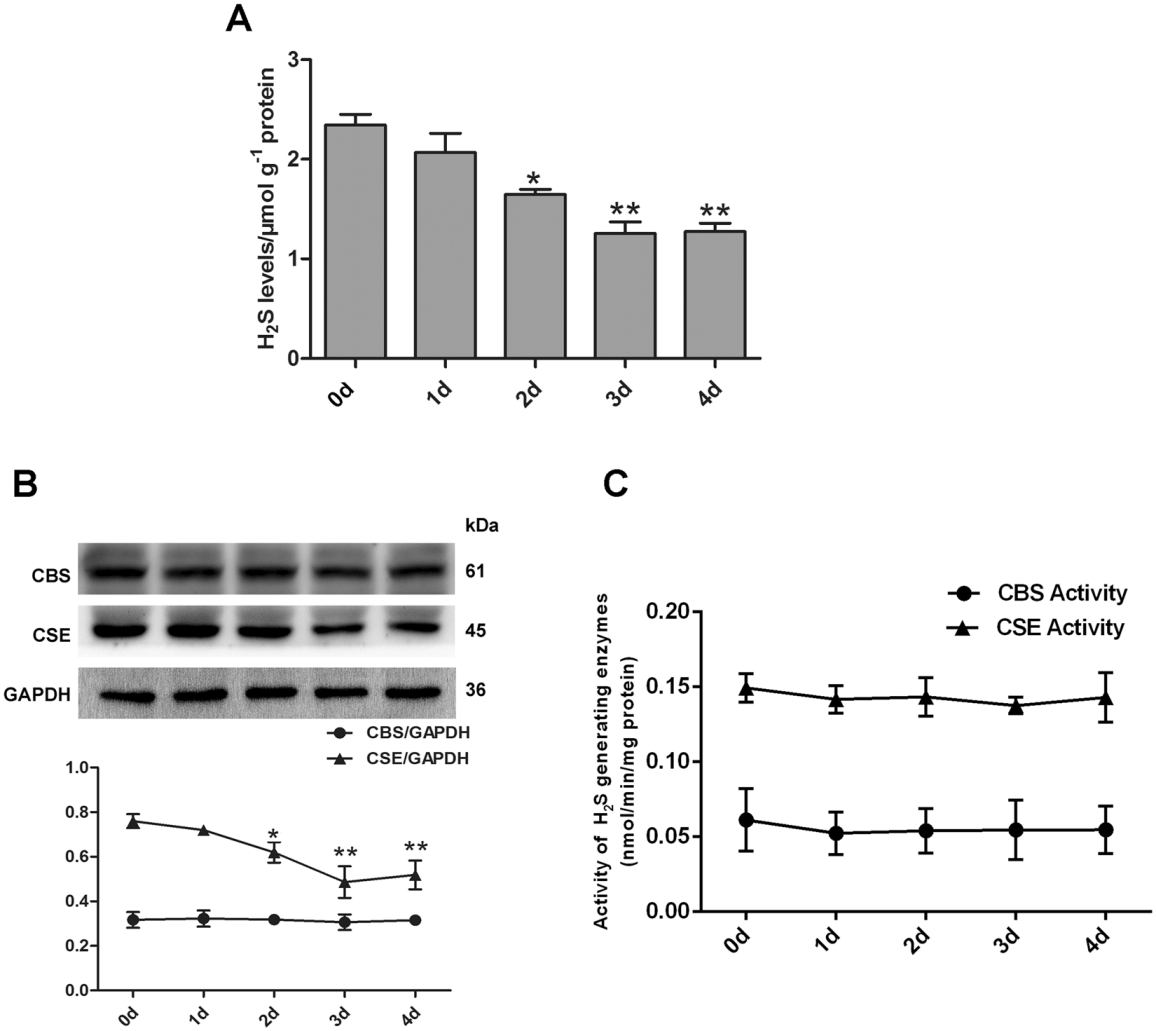
Effect of SM on the sulfide concentration, H_2_S-generating enzymes expression and activity in mouse lungs. Mice were subcutaneously injected with SM (30 mg/kg) to induce lung injury. Sulfide concentrations (**A**) in the mouse lungs were determined using the fluorescent probe RHP-2. CBS and CSE protein expression (**B**) was determined by western blot. CBS and CSE activity (**C**) was measured by enzyme-coupled assay. Data are presented as the mean ± SEM (n = 5). *p < 0.05 vs. CTRL group, **p < 0.01 vs. CTRL group.

To evaluate the role of H_2_S in SM-induced lung injury, we determined the time course of sulfide concentrations after SM treatment. In a previous study, we synthesized and applied the ratiometric fluorescent probe RHP-2 for H_2_S detection^19^. The values obtained by RHP-2 suggested that RHP-2 is suitable for the determination of endogenous sulfide in biological tissues. Using RHP-2, we found that treatment of mice with SM caused a mild decrease as early as 1 day, and the largest decrease was observed between 1 and 3 days (Fig. 1A). Given the reduction of H_2_S levels in SM-treated mouse lungs, we next examined whether H_2_S-generating enzymes were involved in the reduction of H_2_S. As shown in Fig. 1B, CBS protein levels remained constant from 1 to 4 days. However, CSE protein levels were significantly reduced compared with the control group, especially at 3 days. Taken together, SM significantly reduced sulfide concentrations in mouse lungs.

### Functional significance of H_2_S in SM toxicity in mouse lungs

**Figure 2:**
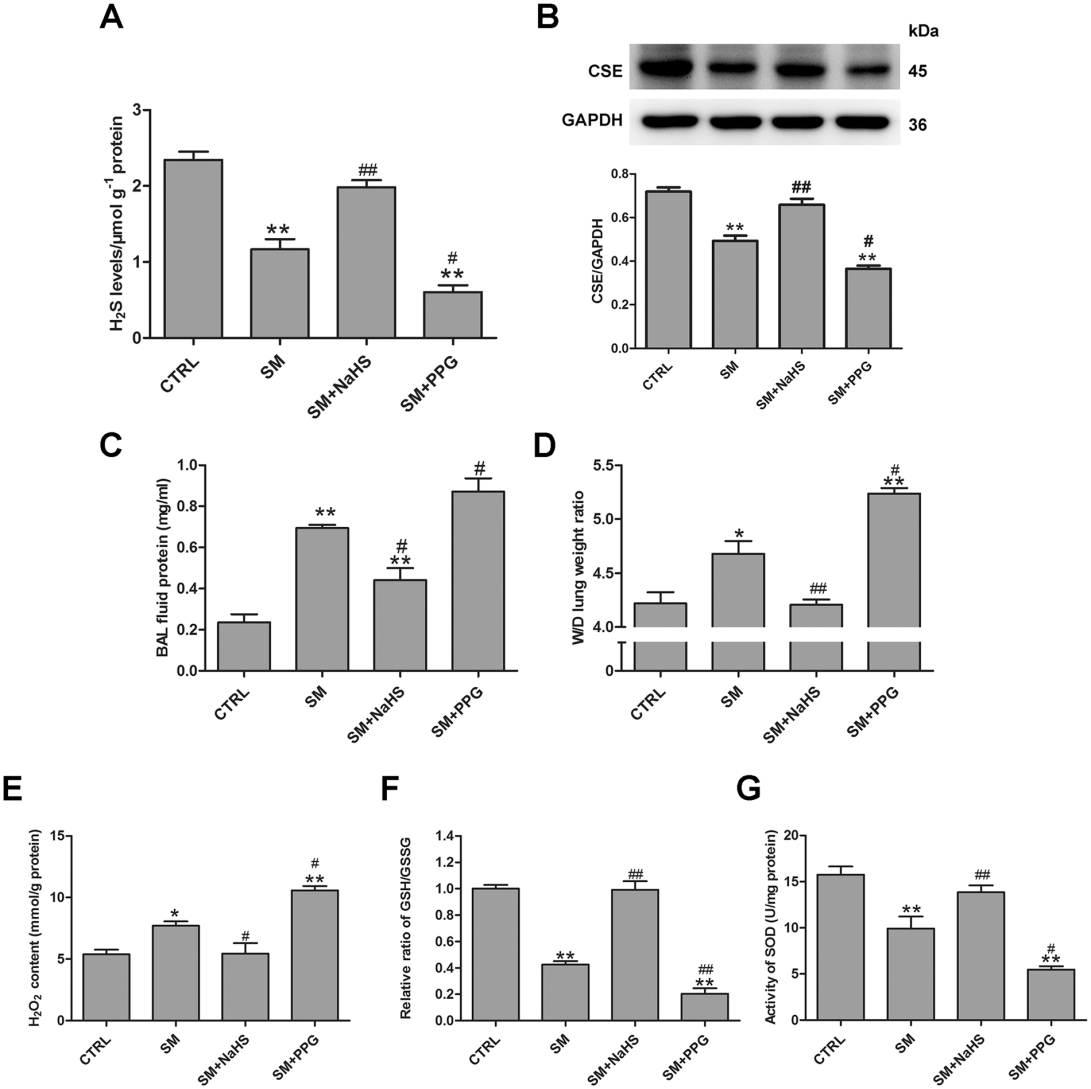
Inhibition of H_2_S reduction protects against SM-induced lung injury. Mice were subcutaneously injected with SM (30 mg/kg), with or without intraperitoneal administration of NaHS (5 mg/kg) or intraperitoneal administration of PPG (30 mg/kg), addition NaHS and PPG were given every day for a total of three doses. Sulfide concentrations (**A**) and CSE protein expression (**B**) were determined by RHP-2 and western blot, respectively. Measurement of protein concentration was performed in BAL fluid (**C**). Lung W/D (**D**) was measured as an index of pulmonary edema formation. H_2_O_2_ content (**E**), GHS/GSSG ratio (**F**), SOD activity (**G**) in mouse lungs were measured as oxidative stress parameters. Data are presented as the mean ± SEM(n = 5). *p < 0.05 vs CTRL group; **p < 0.01 vs CTRL group; ^#^p < 0.05 vs SM group; ^##^p < 0.01 vs SM group.

To evaluate wheather the changes in endogenous sulfide concentrations were the cause or merely a consequence of SM-induced lung injury, experiments were designed to change endogenous sulfide concentrations in mouse lungs. Administration of NaHS targeting endogenous sulfide concentrations resulted in a significant increase in SM treatment mice (Fig. 2A). Although NaHS only partially increased sulfide concentrations, it completely prevented CSE protein downregulation subsequent to SM (30 mg/kg) exposure at 3 days (Fig. 2B). In contrast, DL-propargylglycine (PPG, a CSE inhibitor)-treated mice exhibited markedly reduced endogenous sulfide concentrations and CSE expression compared with SM treatment mice (Fig. 2A,B).

Measurement of the lung wet/dry weight ratio and bronchoalveolar lavage (BAL) fluid protein demonstrated that treatment with SM resulted in significant lung injury by 3 days (Fig. 2C,D). The lung wet/dry weight ratio and BAL fluid protein are indicators of lung injury. Administration of PPG exacerbated lung injuries (Fig. 2C,D). However, preventing the downregulation of sulfide concentrations by treatment with NaHS resulted in significant protection against SM-induced lung injury as revealed by the dramatically reduced W/D ratio and BAL fluid protein (Fig. 2C,D).

**Figure 3:**
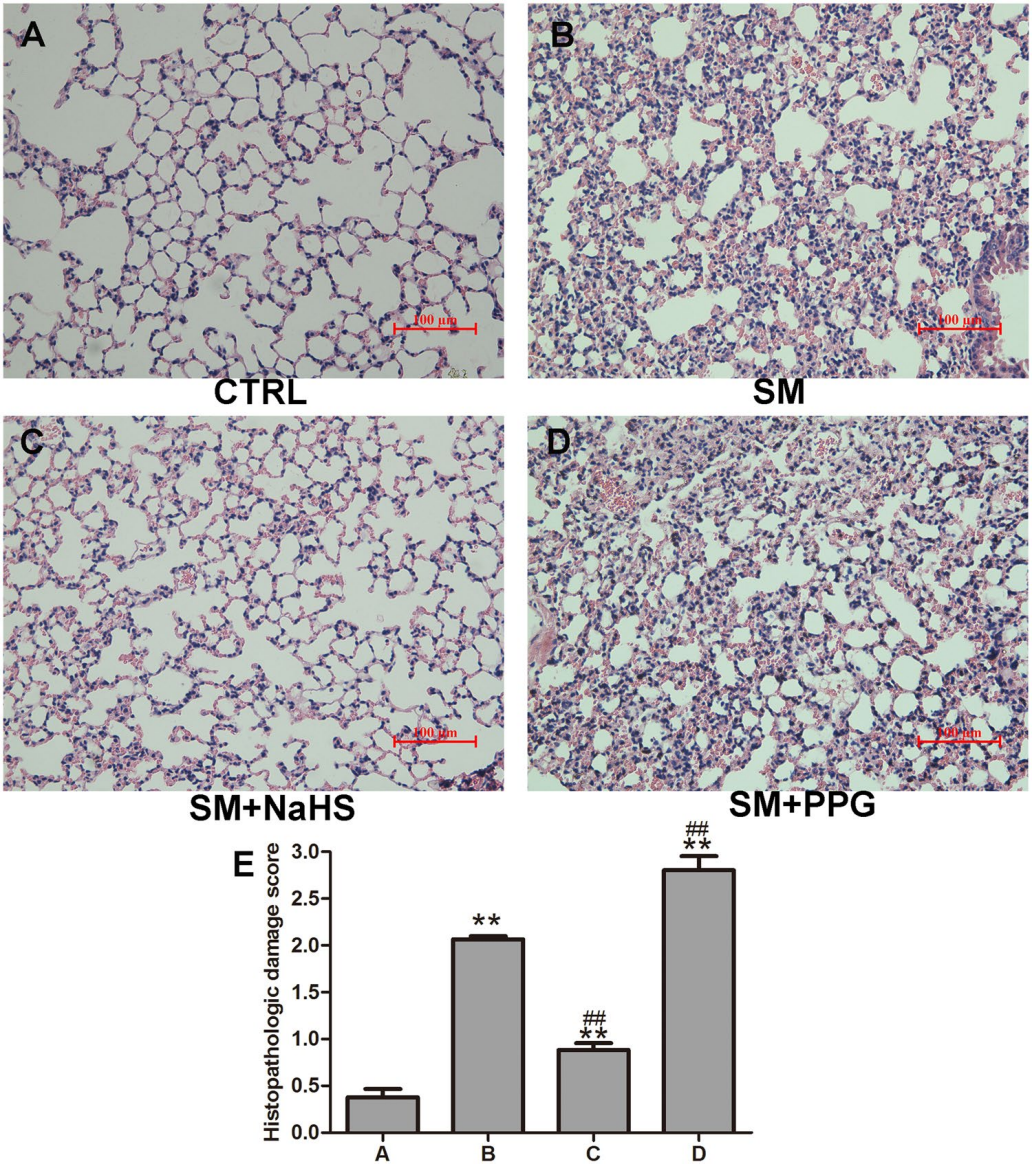
Inhibition of H_2_S reduction improves lung histopathological changes induced by SM. Mice were subcutaneously injected with SM (30 mg/kg), with or without intraperitoneal administration of NaHS (5 mg/kg) or intraperitoneal administration of PPG (30 mg/kg), addition NaHS and PPG were given every day for a total of three doses. The left lower lung was removed for histopathologic examination using hematoxylin and eosin staining. Control (**A**); SM (30 mg/kg) treatment (**B**); SM (30 mg/kg) with NaHS (5 mg/kg) treatment (**C**); SM (30 mg/kg) with PPG (30 mg/kg) treatment (**D**). Original magnification, × 200. Scale bars, 100 μm. The severity of lung injury was scored as described in the Materials and Methods (**E**). Data are presented as the mean ± SEM(n = 4). **p < 0.01 vs control group; ^##^p < 0.01 vs SM group.

In the histopathological study, sections of lung tissue were stained with H&E and scored by histopathological analysis. As shown in Fig. 3B, SM-induced lung injuries were characterized by diffuse interstitial edema, alveolar thickening, marked decreases in alveolar air space, and lung recruitment of leukocytes, with a histopathological damage score of 2.1 ± 0.06 (Fig. 3B,E). These effects were attenuated by NaHS treatment with a significant reduction in the lung injury score 0.9 ± 0.13 (Fig. 3C,E). Figure 3D also demonstrated that administration of PPG aggravated damage in mouse lungs.

To assess oxidant stress, H_2_O_2_ content, the GSH/GSSG ratio, and SOD activity were measured (Fig. 2E–G). NaHS significantly attenuated SM-induced increase in H_2_O_2_ content (Fig. 2E). Furthermore, NaHS also reversed the SM-induced reduction in the GSH/GSSG ratio and SOD activity (Fig. 2F,G). In addition, treatment of mice with PPG significantly increased oxidant stress compared with treatment of mice with SM only (Fig. 2E–G). These data suggest that the changes in sulfide concentrations were not merely a consequence, but the cause of SM-induced lung injury.

### Effects of H_2_S on SM-induced changes in Nrf2 expression, nuclear translocation and the expression of genes downstream of Nrf2 in mouse lung tissues

**Figure 4:**
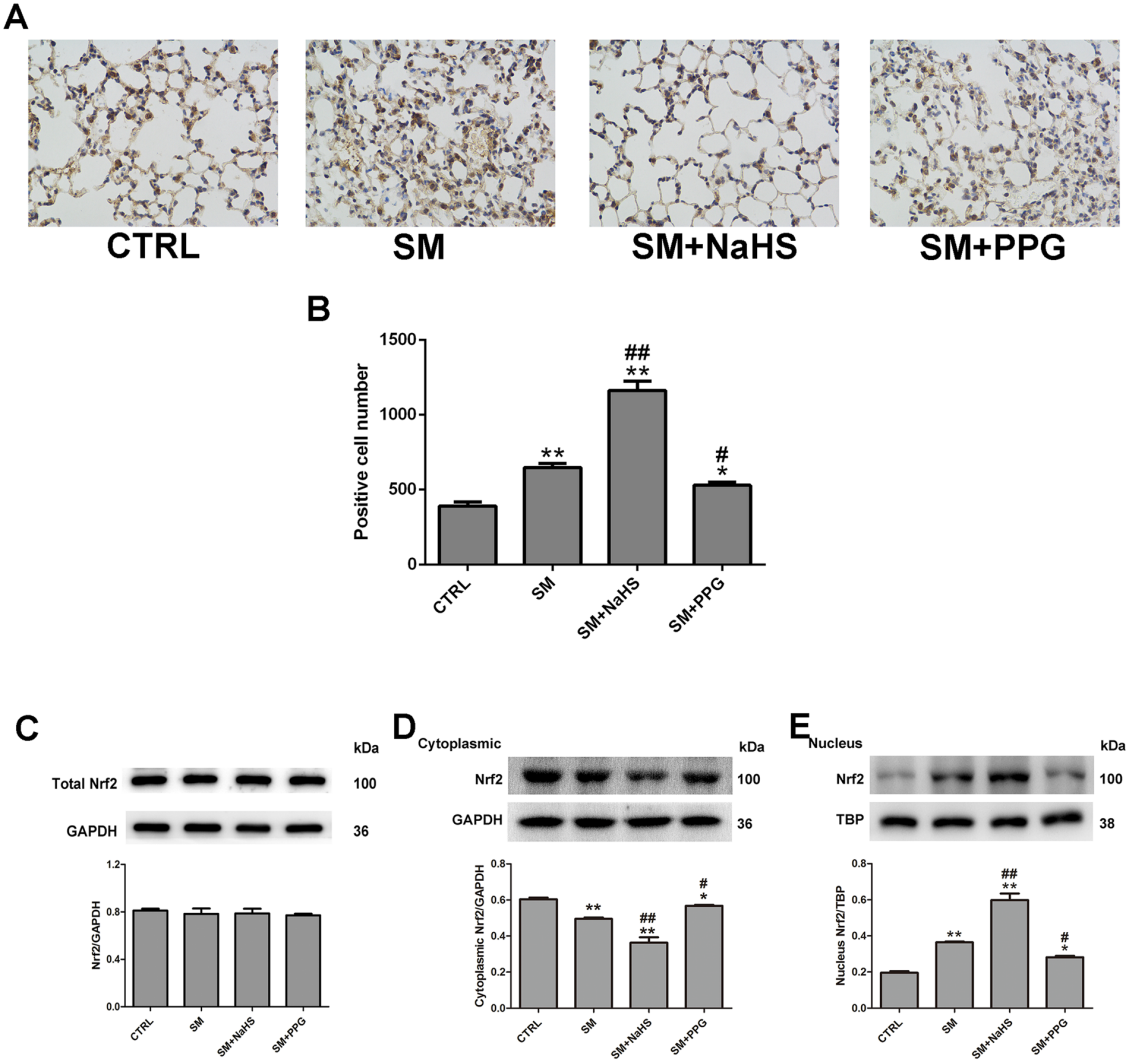
Effects of H_2_S on SM-induced changes in Nrf2 expression, nuclear translocation. Mice were subcutaneously injected with SM (30 mg/kg), with or without intraperitoneal administration of NaHS (5 mg/kg) or intraperitoneal administration of PPG (30 mg/kg), addition NaHS and PPG were given every day for a total of three doses. The left lower lung was removed for Nrf2 protein expression examination using immunohistochemistry (**A,B**). Lung tissues were collected for isolation of cytosolic and nuclear proteins. Total Nrf2 (**C**), Nrf2 cytoplasmic (**D**) and nuclear (**E**) protein levels in lung tissues were determined by western blot analysis. Data are presented as the mean ± SEM (n = 4). *p < 0.05 vs CTRL group; **p < 0.01 vs CTRL group; ^#^p < 0.05 vs SM group; ^##^p < 0.01 vs SM group.

**Figure 5:**
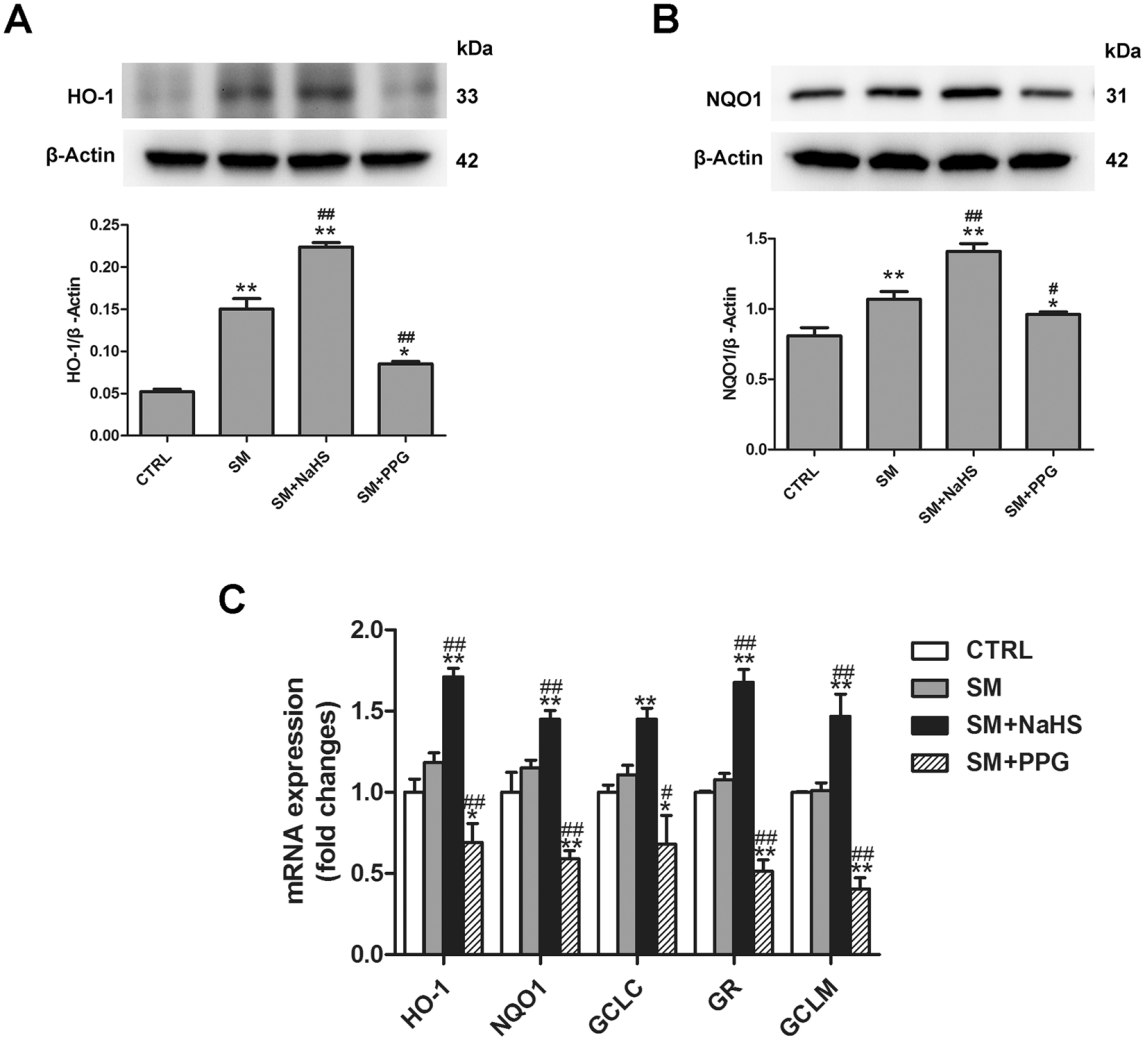
Effects of H_2_S on SM-induced changes in the expression of Nrf2 downstream genes in mouse lung tissues. Mice were subcutaneously injected with SM (30 mg/kg), with or without intraperitoneal administration of NaHS (5 mg/kg) or intraperitoneal administration of PPG (30 mg/kg), addition NaHS and PPG were given every day for a total of three doses. The protein levels of Nrf2 downstream including HO-1 (**A**) and NQO1 (**B**). The mRNA levels of Nrf2 downstream target cytoprotective genes including HO-1, NQO1, GCLC, GR, and GCLM in lungs tissues were determined by RT-PCR (**C**). Data are presented as the mean ± SEM (n = 4). *p < 0.05 vs CTRL group; **p < 0.01 vs CTRL group; ^#^p < 0.05 vs SM group; ^##^p < 0.01 vs SM group.

Nrf2 is a master regulator of the antioxidant response, and was recently considered as a vital target of H_2_S^20, 21^. We examined whether Nrf2 was involved in the protective effects of H_2_S in SM poisoning. SM, NaHS and PPG had minimal effects on Nrf2 mRNA and protein levels in lung tissues (Fig. 4A–E). However, SM did not promote significant Nrf2 nuclear translocation, which was markedly enhanced when combined with NaHS treatment (Fig. 4E). In contrast, treatment of mice with PPG caused a decrease in Nrf2 nuclear translocation compared with SM treatment (Fig. 4E). We also measured the mRNA levels of five Nrf2 downstream target cytoprotective genes: heme oxygenase-1 (HO-1), NAD(P)H dehydrogenase quinone 1 (NQO1), glutamate cysteine ligase composed of catalytic (GCLC), glutathione reductase (GR), and glutamate cysteine ligase composed of modifier (GCLM). As show in Fig. 5C, administration of SM almost no change in HO-1, NQO1, GCLC, GR, and GCLM mRNA expression in lung tissues, and the significant increase was observed when treated with H_2_S following SM exposure. PPG led to a marked reduction in HO-1, NQO1, Trx1, GCLC, GR, and GCLM mRNA levels in lung tissues compared with SM. HO-1 (Fig. 5A) and NQO1 (Fig. 5B) protein expression changed accordingly with the corresponding mRNA levels. Therefore, H_2_S significantly enhanced Nrf2 nuclear translocation, and stimulated both mRNA and protein expression of Nrf2-targeted downstream genes in SM-treated mouse lungs.

### The protective effects of H_2_S against SM-induced oxidative damage in lung cells

**Figure 6:**
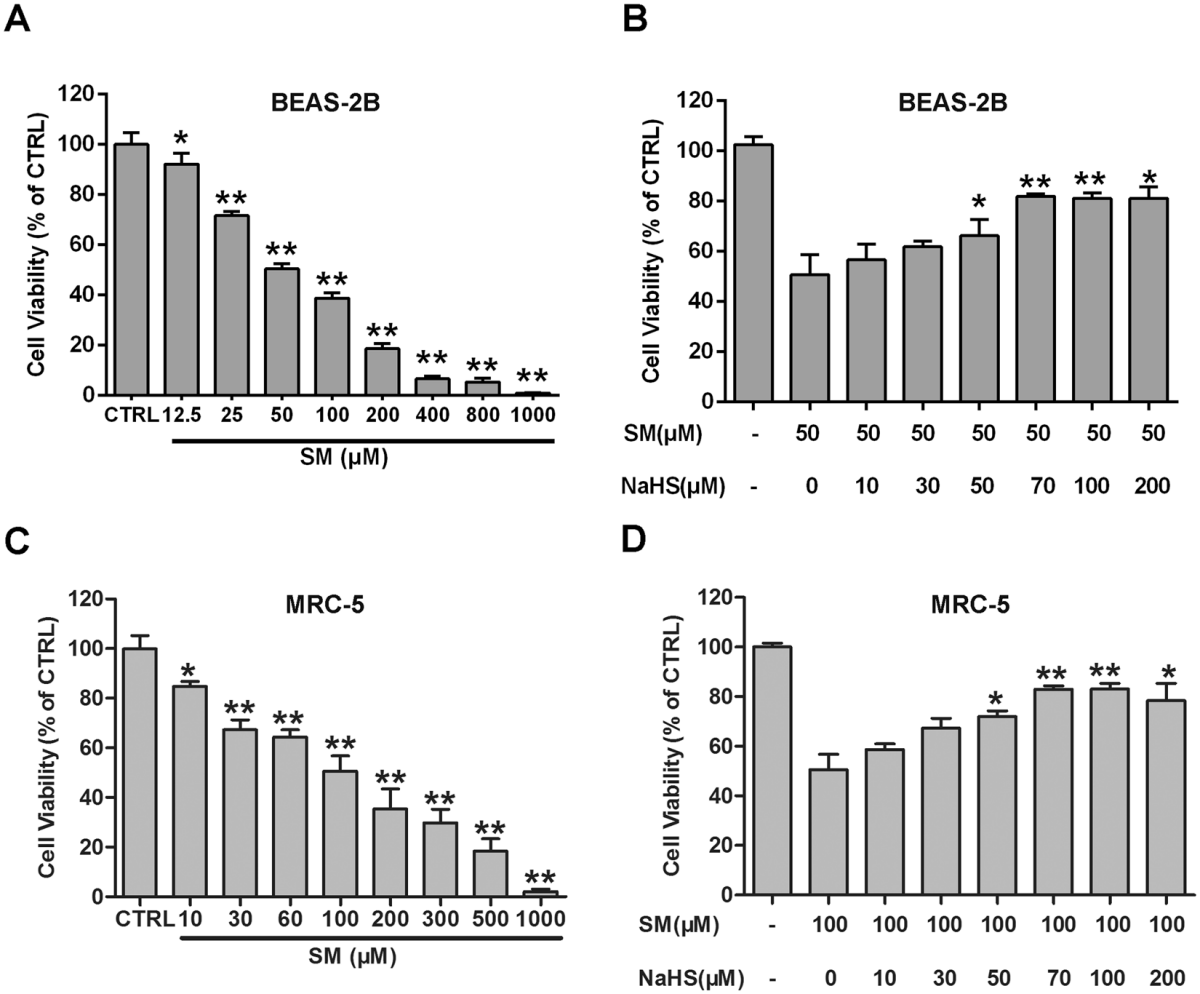
H_2_S protects against SM-induced BEAS-2B cell damage. Untreated or SM-treated BEAS-2B and MRC-5 cells were further cultured for 24 h. Cell viability was assessed using the CCK-8 assay (**A,C**). BEAS-2B and MRC-5 cells were pretreated with the indicated concentrations of NaHS (70 μM) followed by SM (50 μM or 100 μM) treatment. Cells were further cultured for 24 h, and cell viability was tested by the CCK-8 assay (**B,D**). Data are presented as the mean ± SEM (n = 5). *p < 0.05 vs CTRL group (**A,B**); **p < 0.01 vs CTRL group (**A,B**); *p < 0.05 vs SM only group (**C,D**); **p < 0.01 vs SM only group (**C,D**).

The CCK-8 results presented in Fig. 6A,C demonstrated that SM decreased the cell viability of human lung epithelial cells (BEAS-2B) and human fetal lung fibroblast cells (MRC-5) in a does-dependent manner. Notably, NaHS (70 μM) treatment attenuated SM-induced reduction of BEAS-2B and MRC-5 cell viability (Fig. 6B,D) Together, these results demonstrate that NaHS inhibits SM-induced MRC-5 cell death.

**Figure 7:**
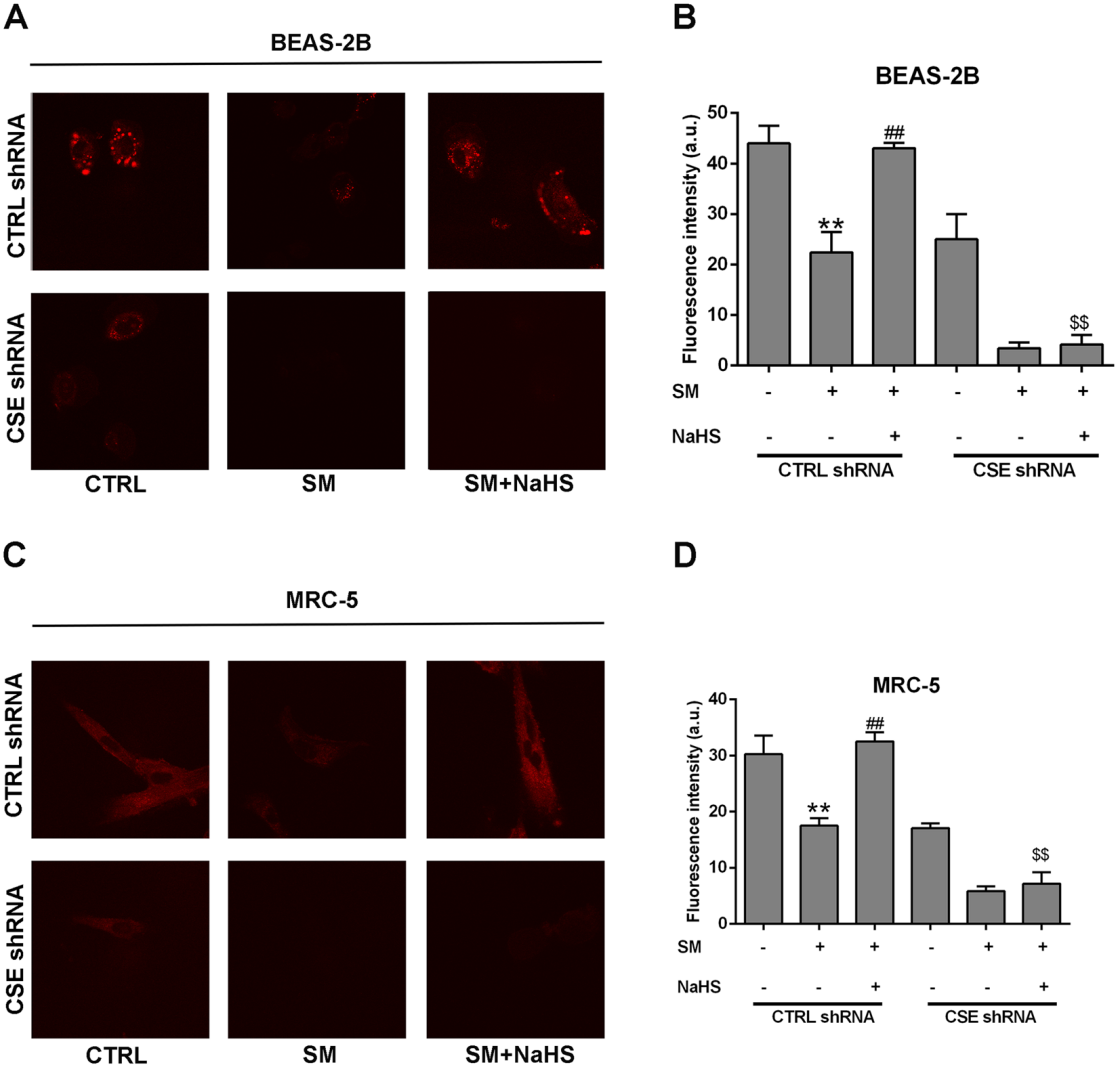
Confocal fluorescence imaging of endogenous H_2_S in living BEAS-2B and MRC-5 cells using NIR-HS. Untreated or SM-treated BEAS-2B and MRC-5 cells were further cultured for 24 h. BEAS-2B Cells were incubated with NIR-HS (5 μM) for 10 min (**A**). MRC-5 Cells were incubated with NIR-HS (5 μM) for 10 min (**C**). The average fluorescence intensity of cells in above images (**G**). Data are presented as the mean ± SEM (n = 5). **p < 0.01 vs A column; ^##^p < 0.01 vs B column; ^$$^p < 0.05 vs C column.

Above, we used the RHP-2 probe to detect H_2_S concentrations in lung tissues *in vitro*. However, for living cell imaging, probes with absorption and emission in the near-infrared (NIR) region are more suitable based on minimal photo damage, deep tissue penetration, and minimum interference from background autofluorescence. Thus, we applied the NIR-HS probe to image endogenous sulfide concentrations. NIR-HS is a synthesized near-infrared fluorescence probe and is effective for this purpose^22^. Cells treated with SM exhibited fainter fluorescence emission compared with control cells (Fig. 7A,C), suggesting reduced endogenous levels of H_2_S in SM-exposed cells. SM-treated cells with NaHS pretreatment exhibited stronger fluorescence compared with the SM group (Fig. 7A,C). Thus, NaHS treatment attenuates the reduced endogenous levels of H_2_S in SM exposed cells. We next constructed cells with CSE knockdown, which exhibited weaker fluorescence compared with control cells, indicating that CSE knockdown reduced endogenous H_2_S levels. When CSE was depleted from SM-treated cells and the cells were then incubated with NaHS, almost no fluorescence emission was observed (Fig. 7B,D). The results indicated that CSE is essential in the regulation of endogenous H_2_S levels and that downregulation of CSE expression reverses the increased endogenous H_2_S levels by NaHS.

**Figure 8:**
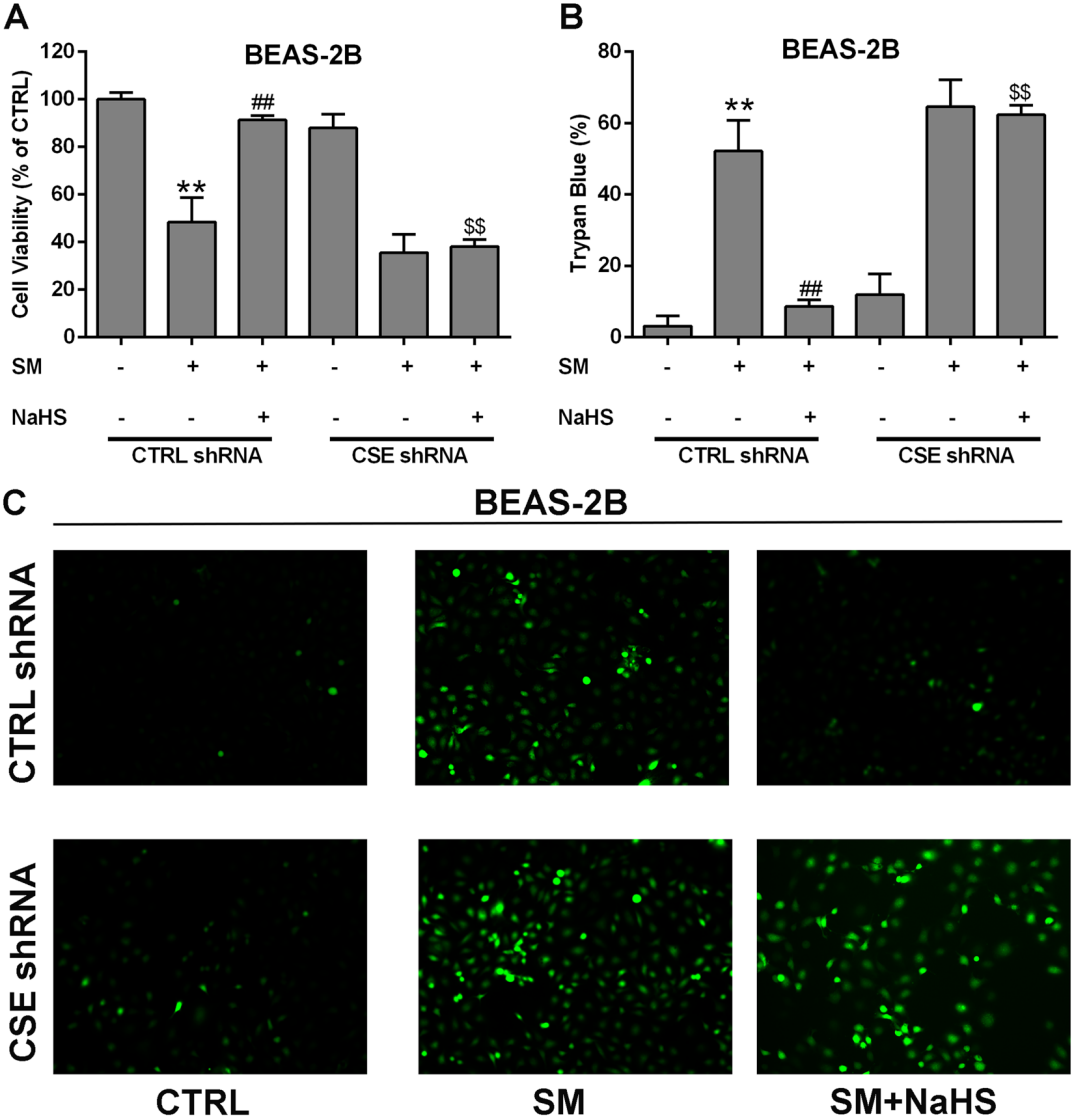
H_2_S protection against SM-induced oxidative damage in BEAS-2B cells. Both wild type and CSE knockdown cells were treated with SM (50 μM), with or without NaHS (70 μM), and cell viability was analyzed by the CCK-8 assay (**A**). Cell death was detected by trypan blue staining (**B**). ROS production was detected by DCFH-DA fluorescence measurement (**C**). Experiments were repeated three times to ensure consistency of the results. Data are presented as the mean ± SEM (n = 5). **p < 0.01 vs CTRL shRNA; ^##^p < 0.01 vs CTRL shRNA with SM; ^$$^p < 0.01 vs CTRL shRNA with SM plus NaHS.

Oxidative stress can be revealed by ROS levels. We measured intracellular ROS levels in BEAS-2B and MRC-5 cells. Dichlorofluorescein diacetate (DCFH-DA) fluorescence intensity was significantly increased in BEAS-2B and MRC-5 cells from the SM group compared with the control group, whereas SM-treated cells with NaHS pretreatment exhibited almost no fluorescence (Figs 8C, S1C). These results imply that SM administration enhances ROS production (Figs 8C, S1C), whereas NaHS pretreatment attenuates the condition. CSE knockdown reversed the reduction of ROS production by NaHS pretreatment (Figs 8C, S1C). In addition, the ROS production results were consistent with the results from the cell viability and trypan blue experiments (Figs 8A,B, S1A,B). The results in this section demonstrate that H_2_S inhibits SM-induced ROS production, and protects SM-treated BEAS-2B and MRC-5 cells via stimulating CSE.

### H_2_S induces Nrf2 nuclear translocation by S-sulfhydrating Keap1 in lung cells

**Figure 9:**
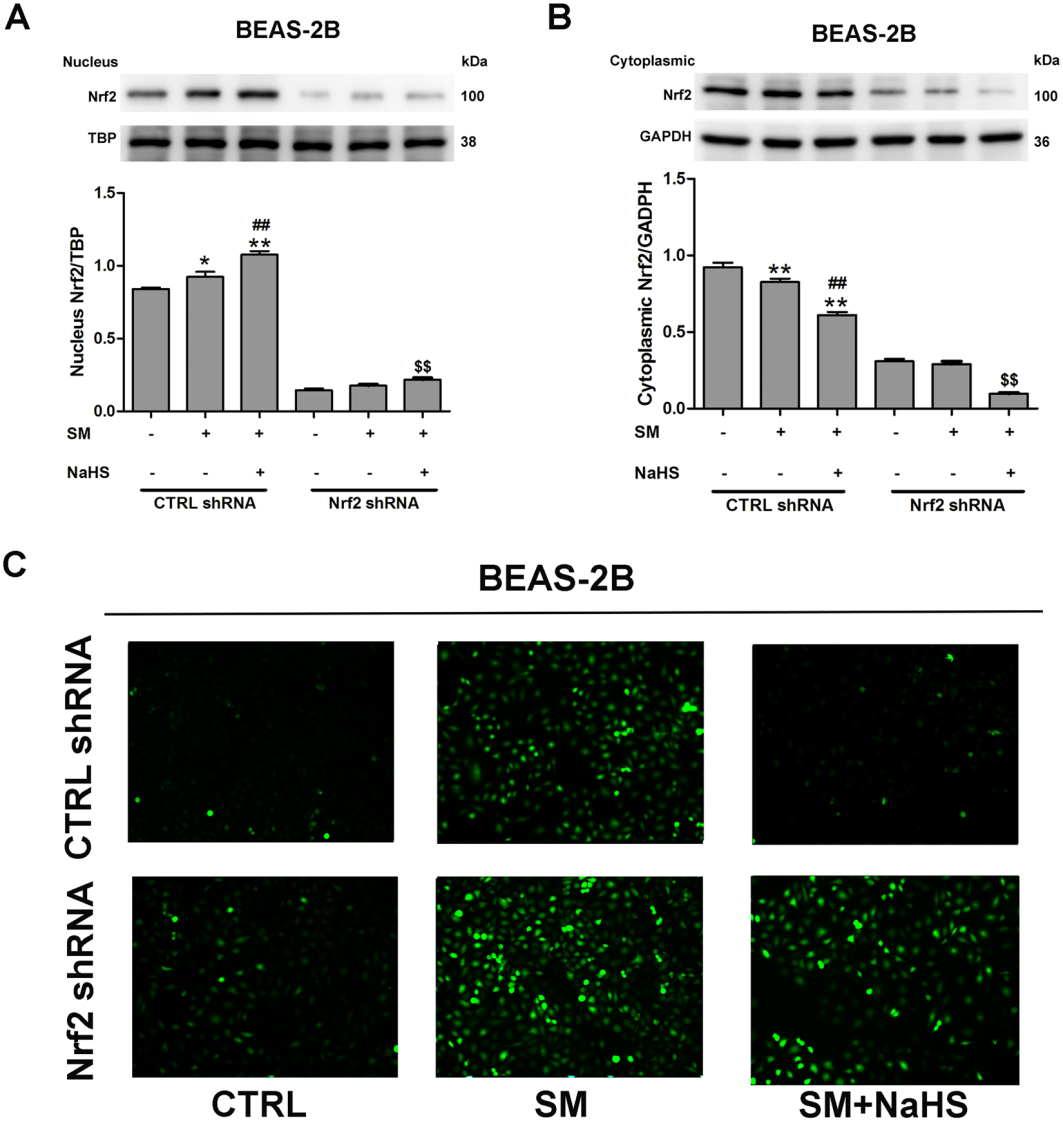
Nrf2 is necessary and sufficient to confer H_2_S benefits against SM-induced oxidative damage. BEAS-2B cells were transfected with control shRNA or Nrf2 shRNA for 48 h and then treated with SM (100 μM) in the presence or absence of NaHS (70 μM) for 24 h. Cells were collected for isolation of nuclear and cytosolic proteins. Nrf2 protein levels in cells were determined by western blot analysis (**A,B**). ROS production was detected by DCFH-DA fluorescence measurement (**C**). Data are presented as the mean ± SEM (n = 5). **p < 0.01 vs CTRL shRNA; ^##^p < 0.01 vs CTRL shRNA with SM; ^$$^p < 0.01 vs CTRL shRNA with SM plus NaHS.

**Figure 10:**
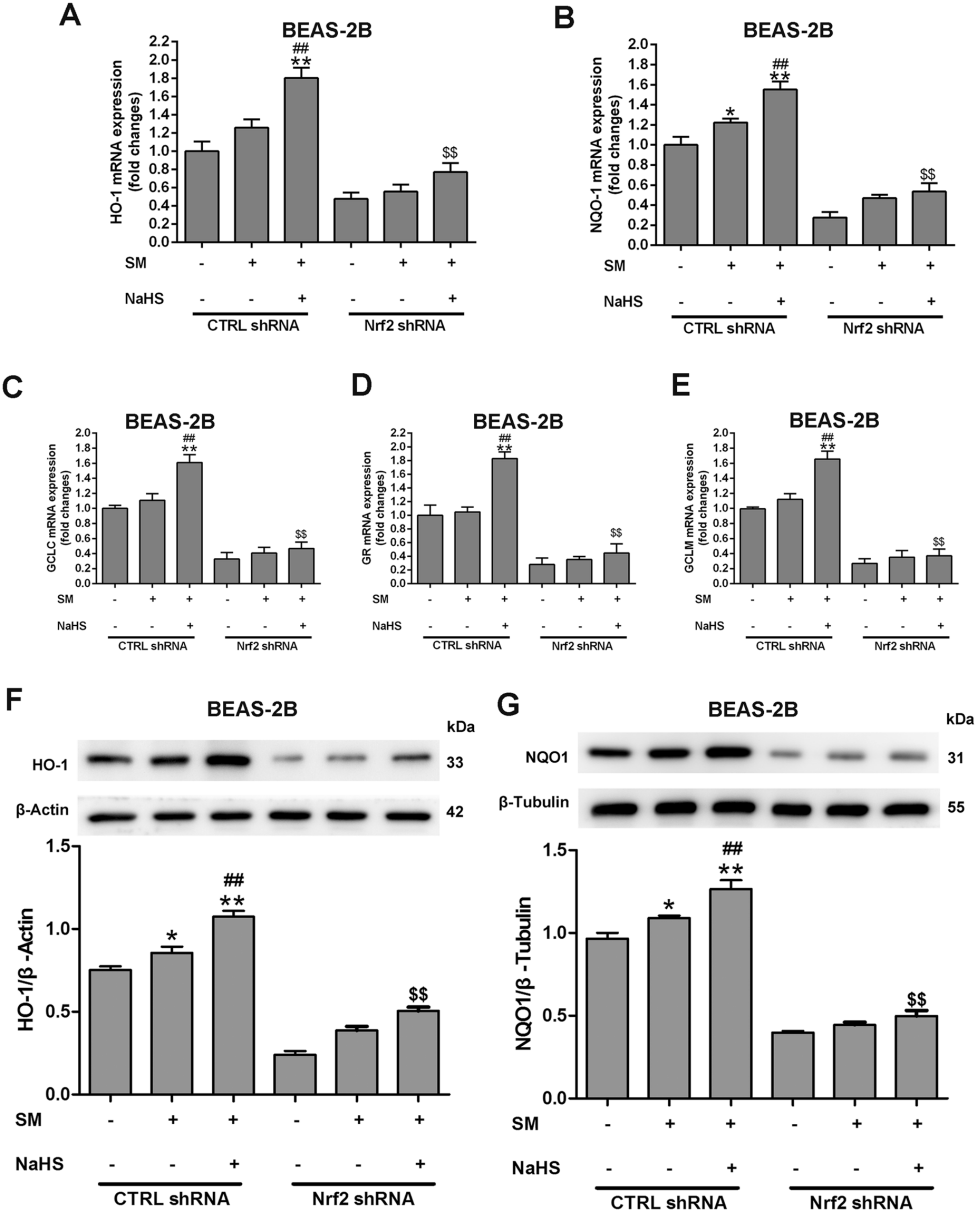
Nrf2 is required for the protective effects of H_2_S against SM-induced oxidative damage. BEAS-2B cells were transfected with control shRNA or Nrf2 shRNA for 48 h and then treated with SM (100 μM) in the presence or absence of NaHS (70 μM) for 24 h. The mRNA levels of Nrf2 downstream target cytoprotective genes including HO-1 (**A**), NQO1 (**B**), GCLC (**C**), GR (**D**), and GCLM (**E**) in cells were determined by RT-PCR. The protein levels of Nrf2 downstream, including HO-1 (**F**) and NQO1 (**G**) in cells were determined by western blot. Data are presented as the mean ± SEM(n = 5). *p < 0.05 vs CTRL shRNA; **p < 0.01 vs CTRL shRNA; ^#^p < 0.05 vs CTRL shRNA with SM; ^##^p < 0.01 vs CTRL shRNA with SM; ^$$^p < 0.01 vs CTRL shRNA with SM plus NaHS.

We examined whether Nrf2 was involved in the protective effect of H_2_S in SM-induced oxidative stress in BEAS-2B and MRC-5 cells. Western blotting revealed that individual transfection with Nrf2 shRNA successfully reduced Nrf2 protein expression at 48 h post-transfection, compared with control cells. Nrf2 protein nuclear (Figs 9A, S2A) and cytoplasmic (Figs 9B, S2B) was in consistent with the data obtained from mouse lungs. The protective effect of H_2_S against SM-induced ROS production was entirely abrogated in the presence of Nrf2 shRNA (Figs 9C, S2C). We also found that treatment of SM-treated cells with NaHS resulted in a striking increase in the mRNA expression of five Nrf2 downstream target cytoprotective genes, including HO-1, NQO1, GCLC, GR, and GCLM (Figs 10A–E, S3A–E). However, the upregulating effects of H_2_S on these genes were almost completely abolished by downregulation of Nrf2 (Figs 10A–E, S3A–E). HO-1 (Figs 10F, S3F) and NQO1 (Figs 10G, S3G) protein expression was consistent with the data on mRNA levels. These results indicated that Nrf2 is required in the protective effects of H_2_S against SM-induced oxidative damage.

**Figure 11:**
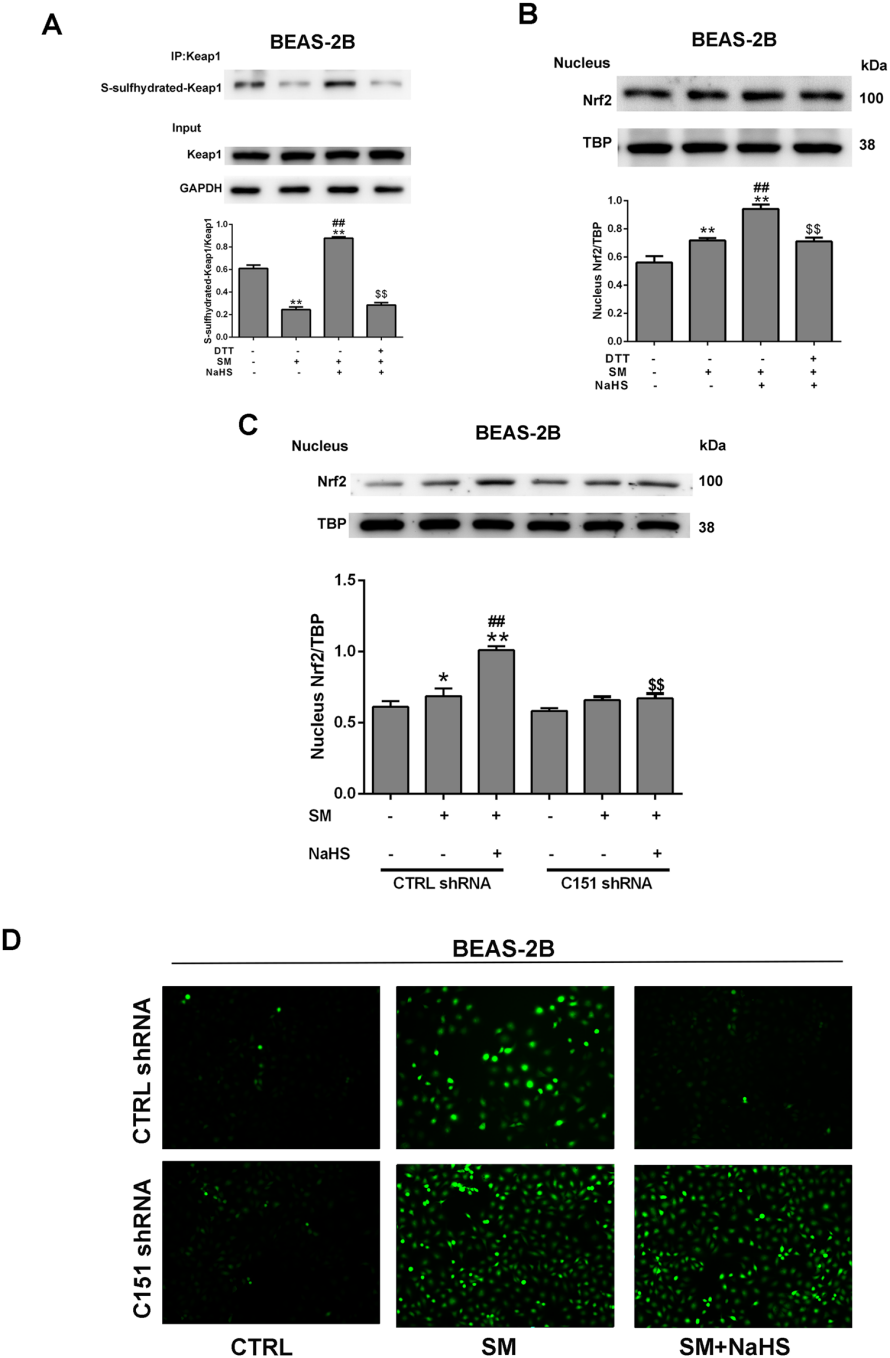
H_2_S S-sulfhydrylates Keap1 to regulate Nrf2 transcription activity in SM-induced BEAS-2B cells. Cells were treated with SM (100 μM), with or without NaHS (70 μM) or DTT (1 mM) pretreatment. S-sulfhydrylation of Keap1 was detected using the “Tag-Switch” method (**A**). Cells were collected for isolation of nuclear proteins. Nrf2 protein levels in cells were determined by western blot analysis (**B,C**). ROS production was detected by fluorescence measurement of the reported DCFH-DA (**D**). Experiments were repeated three times to insure consistency of results. Data are presented as the mean ± SEM (n = 5). *p < 0.05 vs untreated control; **p < 0.01 vs untreated control; ^##^p < 0.01 vs treatment with SM; ^$$^p < 0.01 vs treatment with SM plus NaHS.

Nrf2 is retained in an unactivated state upon binding with Keap1 in the cytoplasm, which serves as an adaptor for the degradation of Nrf2. Certain physiological stimuli activate Nrf2 by disrupting Keap1-Nrf2 interactions and lead to nuclear translocation of Nrf2^23^. S-sulfhydration, which is the addition of one sulfhydryl to the thiol side of a cysteine residue and formation of the persulfide group (R-S-S-H), is a novel post-translational modification by H_2_S. However, the covalent modification by sulfhydration is reversed by reducing agents, such as dithiothreitol (DTT). To further explore the mechanisms of Nrf2 activation in this process, we investigated whether Nrf2 was activated by H_2_S through sulfhydration of Keap1. After incubation with NaHS, MRC-5 cells were treated with SM and subjected to the “Tag-Switch” assay. Keap1 was strongly S-sulfhydrated after NaHS incubation of the cells (Figs 11A, S4A). Moreover, after administration of DTT, NaHS failed to induce S-sulfhydration of Keap1 and subsequent nuclear translocation of Nrf2 in BEAS-2B and MRC-5 cells (Figs 11A,B, S4A,B). After Keap1 being mutated at Cys151, H_2_S failed to induce Nrf2 nuclear translocation and the subsequent reduction of ROS generation (Fig. 11C,D, S4C,D). These findings suggest that S-sulfhydration of Cys151 in Keap1 is critical for Nrf2 activation in SM treated BEAS-2B and MRC-5 cells.

## Discussions

The objective of this study was to assess the presence and functional significance of H_2_S in SM- in SM-induced damage in mouse lungs and lung cells. Using several different experimental approaches, our data consistently showed that H_2_S is a critical component of the signaling cascade of SM-induced lung injuries, at least during the early injury phase.

Thiol/sulfur compounds are potential therapeutics for SM poisoning. Kevin *et al*.^24^ reported that pretreatment of the endothelial cells with the thiol compound *N*-acetyl-L-cysteine selectively prevented apoptotic death induced by SM. The study of Amir *et al et al*.^25^ revealed that extracellularly added glutathione increased cell viability after SM treatment. They also found that GSH depleted cells were more sensitive to SM compared with normal cells, and were also protected by addition of GSH to the growth medium; however, the intracellular GSH content remained low^25^. Wilde found that pretreatment with cysteine esters at concentrations greater than 1 mM protected against the IC_50_ of SM^26^. Rodgers *et al*.^11^ also proposed that thiosulfate and N-acetyl-L-cysteine have possible antidotal effects on the basis of animal studies and analogy from their use in chemotherapy to protect against the toxic side effects of alkylating agents. Hydrogen sulfide (H_2_S) is the metabolite of *L*-cysteine and the precursor of thiosulfate in the human body, which is also one of the thiol/sulfur compounds^27^. As a thiol/sulfur compound, the beneficial effects of H_2_S have been well understood, which could be attributed to its antioxidant effect^12^. Despite a growing number of studies focusing on the beneficial effects of H_2_S, the role of H_2_S in SM poisoning has not been investigated.

The accurate and selective detection of H_2_S is particularly essential for studying its functions in this research. Fluorescence detection methods have become useful tools to explore the physiological roles of H_2_S^28^. In previous studies, we synthetized various novel fluorescent probes including SFP-3, RHP-2 and NIR-HS^19, 22, 29^. Both RHP-2 and NIR-HS were selected and applied in this experiment to detect sulfide concentrations in mouse lungs and lung cells. We applied the ratiometric fluorescent probe RHP-2 to accurately determine sulfide concentrations of mice lungs after SM treatment^19^. Further, we desired to image H_2_S in living cells after SM treatment. However, fluorescent probes with absorption and emission in the near-infrared (NIR) region are more desirable for *in vivo* imaging given the minimum interference from background autofluorescence and minimal photo damage^30^. Here, we applied the near-infrared fluorescent probe NIR-HS to image H_2_S in cells after SM treatment, and NIR-HS works well for this purpose.

Using RHP-2 and NIR-HS, we found that sulfide concentrations in mouse lungs and lung cells were markedly reduced after SM treatment. To the best of knowledge, we are the first group to obtain preliminary data indicating that decreased levels of endogenous H_2_S might play an important role in SM-induced lung injuries. Further investigation revealed that the decrease is closely related to the downregulation of CSE expression after SM treatment. Decreased endogenous H_2_S may contribute to the severe impairment during chronic obstructive pulmonary disease, asthma, pulmonary fibrosis, hypoxia-induced pulmonary hypertension, lung ischemia-reperfusion injury, and acute lung injury^31^. Thus, upregulation of sulfide concentrations in mouse lungs and cells is an important issue. Many researchers have reported that administration of NaHS leads to increased sulfide concentrations in cells or animals^32, 33^. In a previous study, we also found that NaHS attenuates reduction in H_2_S concentrations in the mouse hippocampus in a chronic unpredictable mild stress (CUMS)-induced depression model^19^. NaHS is a short-lived H_2_S donor and can be entirely metabolized in a short time^34, 35^. To avoid the influence of continuous exogenous H_2_S, we chose the short-lived H_2_S donor NaHS rather than the long-lived H_2_S donors, such as GYY4137^36^, SPRC^37^, and ACS 14^38^. In the present experiment, administration of NaHS appeared to attenuate the decrease in sulfide concentrations after SM treatment.

Surprisingly, administration of NaHS resulted in a positive feedback for the enhanced expression of CSE. However, these results are consistent with several recent publications on the upregulation of CBS/CSE expression by exogenous sulfide^32, 33, 39^. In the studies of Zhang *et al*., plasma H_2_S levels and CSE expression in nasal mucosa were decreased in sensitized guinea pigs, and NaHS increased the levels of H_2_S accompanied by upregulation of CSE expression^33^. Han observed that tobacco smoke exposure (TS) reduced CSE and CBS protein expression and the capacity for H_2_S synthesis in mouse lungs, and treatment with NaHS attenuated TS-induced downregulation of CSE and CBS expression^32^. Our previous results also showed that treatment with NaHS significantly attenuated the reduction of CBS expression in the mouse hippocampus in a CUMS-induced depression model^19^. In the present study, we also found that CSE knockdown profoundly inhibited the effects of NaHS against the SM-induced decrease of H_2_S levels *in vitro*. In summary, the increase in endogenous sulfide mainly results from the upregulation of CBS/CSE expression induced by NaHS.

In the present study, we found that SM significantly increased ROS and decreased anti-oxidative enzymes in mouse lung tissues and lung cells. These results are consistent with several references on enhanced oxidative stress by SM. SM accelerates oxidative stress through an increase in endogenous reactive oxidative species (ROS) generation or a decrease in antioxidant capabilities and oxidative DNA repair^40^. Glutathione (GSH) is the key antioxidant element and oxyradical scavenger. SM easily reacts with GSH to produce SM-GSH metabolites which deplete cellular GSH and increase intracellular ROS and other oxidative stress markers^41, 42^. SM also induces lipid peroxidation and protein oxidation. The former generates highly reactive electrophilic lipid peroxidation end products, whereas the latter modifies the functional activity of enzymes and structural proteins^43, 44^. Therefore, SM toxicity may be the result of direct damage induced by oxidative stress. The antioxidant effects of H_2_S have been well established, particularly with respect to lung injuries. In the study of Arul *et al*., the H_2_S donor reduced ROS in an experimental model of O^2–^induced neonatal lung injury^16^. Moreover, Roland reported that Na_2_S (also a short-lived H_2_S donor) represents a novel therapeutic agent to prevent both pulmonary glutathione depletion and ventilator-induced lung injury by activating Nrf2^45^. Wei *et al*.^46^ investigated the modulatory effect of NaHS on lung tissue-oxidized glutathione and total antioxidant capacity (T-AOC) in the development of hypoxic pulmonary hypertension (HPH). Treatment with NaHS decreased GSSG and increased T-AOC levels in lung tissues. Consistent with these observations, our study showed that elevated endogenous H_2_S significantly reversed oxidative stress injury induced by SM. Additionally, we found that inhibition of endogenous H_2_S aggravates oxidative stress injury induced by SM.

Nrf2 is a critical redox sensor and is one of the key regulators of antioxidant responses^47^. Under normal and unstressed circumstances, Nrf2 is mostly found in the cytosol bound to Keap1, which promotes Nrf2 ubiquitination and degradation via a Cul3-E3-dependent mechanism^48^. Nrf2 is dissociated from Keap1 directly through post-translational modulation of crucial cysteine residues in Keap1 protein^49^. After dissociation from Keap1, Nrf2 translocates to the nucleus and binds to promoters containing the ARE sequence, activating the transcription of antioxidant genes and proteins such as HO-1, NQO1, GCLM, GR, and GCLC^50^. Nrf2 has emerged as an important target in lung injury caused by endotoxin, paraquat, bleomycin, and hyperoxia^51^, but the role of Nrf2 in SM-induced lung injury remains unknown. In the present study, slight increases in Nrf2 and downstream protein expression were observed in lung tissues exposed to SM. No obvious changes in Nrf2 nuclear translocation and protein expression in lung tissue were observed, suggesting that administration of SM had no significant influence on Nrf2 signalling cascades. However, administration of NaHS exhibited a stimulatory effect on Nrf2 nuclear translocation and expression of Nrf2 downstream proteins in lung tissue, suggesting that NaHS activates Nrf2 signalling cascades after SM treatment. Xiang *et al*.^52^ reported that H_2_S induces the nuclear accumulation of Nrf2 in lung tissue and consequently up-regulates the expression of antioxidant genes in smoking rats. In the study of Laura *et al*., NaHS up-regulated Nrf2 nuclear translocation, and the transcription of the two key downstream antioxidant genes Peroxiredoxin-1 and NAD(P)H dehydrogenase quinone 1^53^. In our study, similar to H_2_S activation of Nrf2 signaling cascades *in vivo*, administration of NaHS significantly stimulated Nrf2 nuclear translocation *in vitro*. In addition, Nrf2 knockdown profoundly inhibited the protective effects of H_2_S against SM-induced oxidative damage *in vitro*. Taken together, these findings suggest that the activation of the Nrf2 signaling cascade might explain the attenuation of SM-induced lung injury exerted by H_2_S.

A widely accepted method for Nrf2 nuclear accumulation suggests that modification of the Keap1 cysteines directly leads to the dissociation of the Keap1-Nrf2 complex^54^. Recently, Yang suggested that H_2_S S-sulfhydrates Cys151 in Keap1, which stimulates the dissociation of Nrf2 to enable its translocation to the nucleus^55^. Xie also demonstrated that H_2_S attenuates diabetes-accelerated atherosclerosis, which may be related to inhibition of oxidative stress via Keap1 sulfhydrylation to activate Nrf2 signaling^56^. We found that H_2_S promotes S-sulfhydrated Keap1 in SM-treated lung cells, demonstrating that Keap1 S-sulfhydration plays a significant role of in the protective effects of H_2_S against SM-induced lung injuries.

It could not be omitted that the vitro model applied in this study could not perfectly simulate the vivo model. Once absorbed, intact SM could be systemically distributed to almost all organs and tissues including the kidneys, liver, intestines, and lungs. Also, a significant accumulation of intact SM was observed in adipose tissues and that SM in adipose tissues would be released to different organs, including lungs^57^. Thus, subcutaneously injections of SM could cause injury to lung directly. However, subcutaneously injections of SM could also cause injury in other tissues and organs, which lead to the release of secondary mediators. Subcutaneously injections of SM could cause both direct and indirect lung injury *in vivo* studies. While in the vitro studies, SM was directly added to cell culture media, which is merely direct injury.

In summary, our data identified H_2_S as a critical actor in SM-induced lung injuries, although its role has been neglected for a long time. Its role deserves deep and thorough investigation to understand the context of the mechanism. Providing proper levels of endogenous H_2_S could serve as a promising therapeutic measure for the treatment of SM-induced injuries.

## Methods

### Animals and administration

All animal experiments were performed in accordance with the guidelines approved by the Institutional Animal Care and Use of Second Military Medical University on the use and care of laboratory animals. Adult male ICR mice, purchased from the Experimental Animal Centre of Second Military Medical University, weighing 20–25 g were used. Before the experiment, mice were acclimated for 1 week. The mice were randomly divided into every group (n = 8 each): the control group, without treatment; the model group, subcutaneous injection of SM (30 mg/kg); the NaHS group, intraperitoneal injection of NaHS (5 mg/kg) 30 min after the subcutaneous injection of SM, NaHS were given once a day for a total of three times; and the PPG group, intraperitoneal injection of PPG (30 mg/kg) 30 min after the subcutaneous injection of SM, PPG were given once a day for a total of three times. On the day 3, all surviving experimental animals were sacrificed. The chest were opened and lungs were collected for analysis. Part of the lung tissues was snap frozen with liquid nitrogen and stored at -80 °C until analysis, the other one was fixed in 4% buffered formaldehyde for histopathologic evaluation.

### Determination of sulfide in the mouse lung

Sulfide concentration was measured using RHP-2 as described previously^19^. Briefly, the lung was immediately isolated after the mice were sacrificed and homogenised with 9 volumes (w/v) of ice-cold 100 mM PBS buffer (pH 7.4); the homogenate was centrifuged at 10,000 × g for 10 min at 4 °C. All of the procedures were performed in an ice bath, and the homogenate supernatants were immediately transferred for sulfide measurement. All fluorescence measurements were recorded on a Hitachi F4600 Fluorescence Spectrophotometer. Protein concentrations of the mouse lung were determined with a Pierce BCA Protein Assay Kit. Twenty microlitres of 10% homogenate supernatant (final concentration 2%, w/v), 69 mL of PBS buffer (100 mM, pH 7.4) and DI H_2_O (10, 9, 8, 7 and 6 mL, respectively) was added into Eppendorf tubes. Thereafter, the samples were added 0, 1, 2, 3, 4 mL of Na_2_S stock solution (100 mM) as an internal standard, subsequently they were added 1 μL of 1.0 mM RHP-2 probe (final concentration 10 mM) for incubation. Emission spectra (λ_ex_ = 415 nm) were measured after incubation of the mixture at 37 °C for 40 min. In order to obtain the aero point, 1 mL of 100 mM (final concentration 1 mM) ZnCl_2_ was spiked into the samples to trap H_2_S. The sulfide concentration in each sample was calculated by the calibration curve of Na_2_S, and the results were expressed as μmol g^−1^ protein.

### Bronchoalveolar lavage

Measurements of protein concentration was conducted as previously described^58^. In brief, carefully and slowly instilling and withdrawing PBS (0.5 ml) into the lung through a cannula in the trachea to obtain bronchoalveolar lavage fluid. BALF was centrifuged (300 × g, 8 min).

### Lung Wet-to-dry weight ratio

The lung was weighed immediately after opening the chest to obtain the ‘wet’ weight. The tissues were dried in a 65 °C oven with desiccant for 72 h and weighed again to obtain ‘dry’ weight. The wet/dry weight (W/D) ratio of the lung tissues was then calculated to compare lung water content.

### Oxidative stress biomarkers

Levels of H_2_O_2_ in lungs were measured using a Hydrogen Peroxide Assay Kit (Beyotime Institute of Biotechnology, Shanghai, China). Eppendorf tubes containing 100 μl of test solutions and 50 μl of supernatants were placed at room temperature for 30 min and measured immediately with a spectrometer at 560 nm. Absorbance values were calibrated to a standard curve generated with known concentrations of H_2_O_2_. The intracellular GSH and GSSG levels were determined with GSH and GSSG assay kit (Beyotime Institute of Biotechnology, Shanghai, China). GSSG content was measured by use of the 5,5′-dithiobis(2-nitrobenzoic acid) and GSH (GSSG) recycling system. The amount of GSH was calculated by subtracting the amount of GSSG from total glutathione. The contents of SOD was determined using the Total Superoxide Dismutase Assay Kit with WST-8 (Beyotime Institute of Biotechnology, Shanghai, China) following the manufacturer’s instruction.

### Lung histopathologic examination

Lung tissues were embedded in paraffin and cut into 5 μm sections and then stained with hematoxylin-eosin (H&E) stain. Histologic sections were observed using light microscopy and scored by two pathologists with expertise in lung pathology. According to 6-grade scale, histopathologic parameters were evaluated semiquantitatively compared with controls^23^.

#### Immunohistochemistry

After deparaffinized, tissue sections were antigen retrieved by citrate buffer (10.2 mM sodium citrate, 0.05% Tween 20, pH 6.0) and quenched of endogenous peroxidase in 3% hydrogen peroxide for 15 min. Then the sections were blocked in 3% BSA at room temperature for 30 min. This was followed by incubating with antibody to Nrf2 for overnight at 4 °C. The immunohistochemical staining was completed under the guidance of an EnVision Kit (Dako, Carpinteria, CA).

#### Cell culture and administration

MRC-5 and BEAS-2B cells were purchased from the cell bank of Chinese Academy of Sciences. MRC-5 cells were cultured in MEM media (Hyclone) with the addition of FBS [15% (v/v), Foetal bovine serum, Gibco] and penicillin/streptomycin (100 μg/mL). While BEAS-2B cells were cultured in DMEM media (Hyclone) with the addition of FBS [10% (v/v), Foetal bovine serum, Gibco] and penicillin/streptomycin (100 μg/mL). The condition for cell culture is 5% CO_2_ at 37 °C in an incubator. NaHS was dissolved in MEM media or DMEM media without FBS to the final concentration of 70 μM. The cells were treated with NaHS for 1 h and then exposed to 50 μM (BEAS-2B) or 100 μM (MRC-5) SM dissolved in PBS for 30 min.

### Confocal fluorescence imaging

Sulfide concentrations were determined using NIR-HS as described previously^22^. BEAS-2B and MRC-5 cells were harvested and transferred to a coverglass (Lab-Tek^®^ II Chambered Coverglass, NaleNunc, Naperville, USA) after growing to 80% confluence. A final concentration of 5 μM NIR-HS (1.0 mM stock solution in DMSO) was added to the cell media and incubated at the previous conditions for 10 min. Before imaging, all the cells were washed with PBS buffer for three times. An Olympus FV1000 confocal laser scanning microscope was applied for the confocal fluorescence imaging with ×60 oil objectives. The excitation wavelength was 635 nm. The fluorescence images (660–760 nm) were collected at 1024 × 1024 pixels, and were analyzed with Olympus software (FV10-ASW).

### CCK-8 cell viability assay

The cell viability was assessed using the CCK-8 assay (Beijing Zoman Biotechnology Co., Ltd. Beijing, china). Before proliferation detection, 10 μl of CCK-8 was added to the 100 μl cultured cell. After co-cultured for 1.5 h, Optical Density (OD) values at 450 nm were measured by a microplate reader.

### Trypan blue staining of “dead” lung cells

Cells were stained with 0.4% (diluted by PBS) for 3 min, followed by counting blue cells and total cells under microscope, and then the relative proportion of the dead cells was calculated using these counts.

### Plasmid-shRNA construction and transfection

Lentivirus-shRNA plasmid targeted Nrf2 (human) and CSE (human) were provided by Genomeditech (Shanghai, China). Briefly, BEAS-2B and MRC-5 cells were infected with the Nrf2-shRNA plasmid or the CSE-shRNA plasmid using lipofectamine 3000 (Invitrogen) according to the protocol for transfection, respectively. The expressions of targeted protein and the loading control in stable cells were verified by Western blots.

### ROS Measurement

Intracellular ROS content was measured as the instruction of Reactive Oxygen Species Assay Kit (Beyotime Institute of Biotechnology, Shanghai, China). DCFH-DA was dissolved in dimethyl sulfoxide, and then diluted with culture media into a final concentration of 10 μM. After incubation with 10 μM DCFH-DA at 37 °C for 20 min in the dark, the cells were washed three times with culture media, and then the DCF fluorescent images were taken by a fluorescent microscope.

### Western blots

Cells or tissues were washed with cold PBS, and then lysed with RIPA buffer (1% Triton X-100, 1% deoxycholate, 0.1% SDS) with the adding of protease inhibitors (1 mM PMSF, 20 mM NaF, 1 mM NaVO_3_). The concentrations of proteins were measured by BCA protein assay kits. For western blot analysis, the extracted protein samples were separated by 10% SDS-polyacrylamide gel electrophoresis (SDS-PAGE), and the protein samples were then transferred to polyvinylidine difluoride membranes (PVDF; Millipore, Whatman). 5% Skim milk powder was used for blocking the membranes in a washing buffer for 2 h at 25 °C. The blocked membranes were immersed in the solution of primary antibodies and incubated overnight at 4 °C. After rinsing, each membrane was incubated with secondary antibodies conjugated with horseradish peroxidase, followed by visualization with enhanced chemiluminescence reagent (ECL Western Blotting System; Amersham Bioscence). Protein bands were scanned and quantified by densitometric analysis using Image J version 1.34 s software.

### S-sulfhydration assay

H_2_S S-sulfhydration was assessed as described previously^59^. Summarily, the protein of Keap1 was pulled down by immunoprecipitation, followed by treating with biotin-linked cyanoacetate. Samples were resuspended in Laemmli buffer, heated, and subjected to western blot analysis using anti-biotinantibody. The biotinylated proteins were eluted by an SDS-PAGE gel and subjected to western blotting analysis using anti-Keap1 antibody.

### Reverse transcriptase-PCR (RT-PCR)

Trizol reagent (Invitrogen Life Technologies) was applied for the extraction of total RNA. DNA-free total RNA was used to perform the reverse transcription with the Revert Aid First Strand cDNA Synthesis Kit (Thermo). The transcribed cDNA was utilized for PCR amplification with specific primers summarized in previous publication. The primers for HO-1 were: forward 5′-ACAGCATGTCCCAGGATTTGTC-3′ and reverse 5′-GGAGGCCATCACCAGCTTAAAG-3′; the primers for NQO-1 were: forward, 5′-CCATTCTGAAAGGCTGGTTTG-3′, and reverse: 5′-CTAGCTTTGATCTGGTTGTC-3′. the primers for GCLC were: forward 5′-TTACCGAGGCTACGTGTCAGAC-3′ and reverse 5′-TGTCGATGGTCAGGTCGATGTC-3′; the primers for GR were: forward 5′-TGTTTTGCTCCTGATCTGA-3′ and reverse 5′-TCGGGGAATTCAATACTCA-3′; the primers for GCLM were: forward 5′-AATCAGCCCTGATTTGGTCAGG-3′ and reverse 5′-CCAGCTGTGCAACTCCAAGGAC-3′. Glyceraldehyde-3-phosphate dehydrogenase (GAPDH) was tested as an internal control.

### Statistical analyses

Values were expressed as the mean ± SEM. All statistical analysis were performed by one-way ANOVA using SPSS software, version 16.0 (SPSS Inc., Chicago, IL). Significance was chosen if p < 0.05.

## Electronic supplementary material

Supplementary Information
