# Supplementary material for: Neglected role of hydrogen sulfide in sulfur mustard poisoning: Keap1 S-sulfhydration and subsequent Nrf2 pathway activation
Source: Sci Rep. 2017 Aug 25;7:9433. doi: 10.1038/s41598-017-09648-6 (PMC5572733; doi:10.1038/s41598-017-09648-6)
Supplement: Supplementary file 1 — Supplementary Information [file 41598_2017_9648_MOESM1_ESM.pdf]

Neglected role of hydrogen sulfide in sulphur mustard poisoning: Keap1 S-sulfhydration and subsequent Nrf2 pathway activation

Wenqi Meng[1,*], Zhipeng Pei[1,*], Yongwei Feng[1,*], Jie Zhao[1], Yongchun Chen[1], Wenwen Shi[1], Qingqiang Xu[1], Fengwu Lin[2], Mingxue Sun[1] & Kai Xiao[1]

[1] Lab of Toxicology & Pharmacology, Faculty of Tropical Medicine and Public Health, Second Military Medical University, Shanghai 200433, China.

[2]China-Japan Union Hospital of Jilin University, Changchun 130033, China.

*These authors contributed equally to this work.

Correspondence and requests for materials should be addressed to K.X. (kaixiaocn@163.com), or M.S. (sunmx1985@163.com)

The supplementary Figures of the manuscript

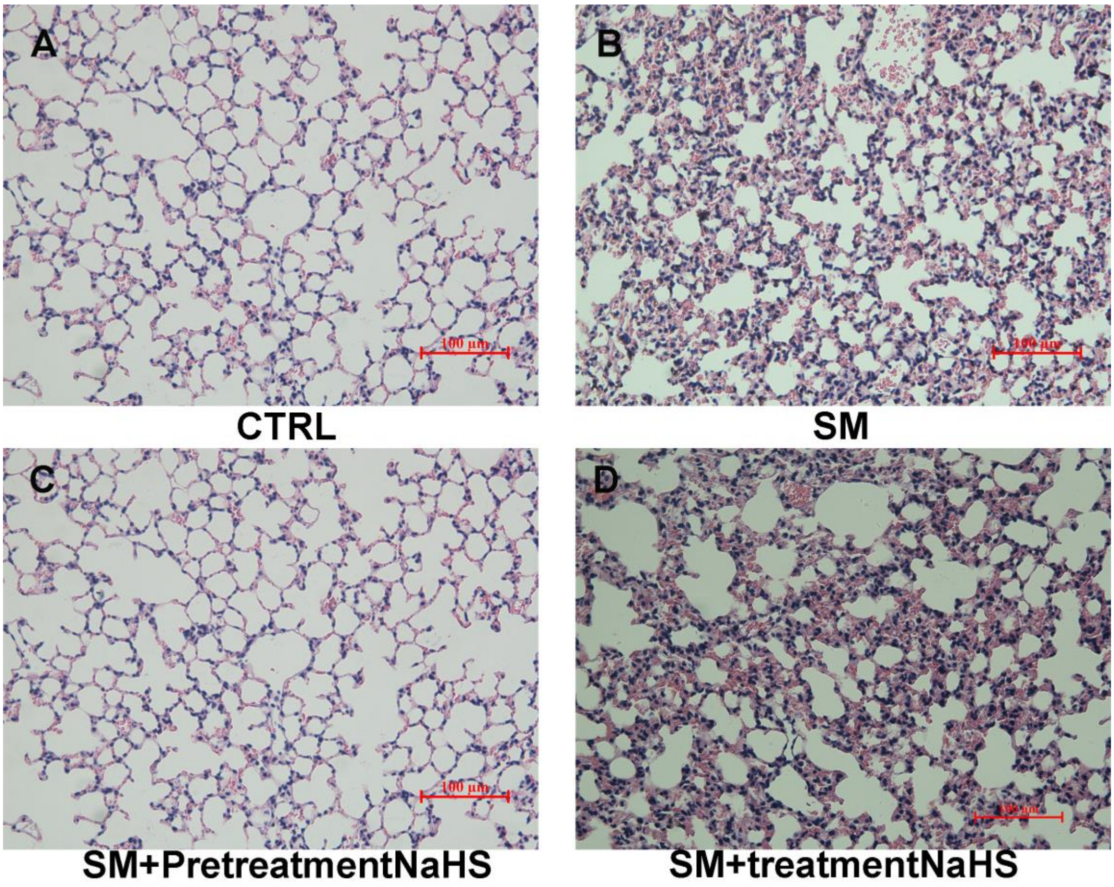

Supplementary **Fig. 1.** Mice were subcutaneously injected with SM (30mg/kg), with or without intraperitoneal administration of NaHS (5 mg/kg). The left lower lung was removed for histopathologic examination using hematoxylin and eosin staining. Control (A); SM (30 mg/kg) treatment (B); SM (30 mg/kg) with NaHS (5 mg/kg) pretreatment (C); SM (30 mg/kg) with NaHS (5 mg/kg) treatment (D). Original magnification, ×200. Scale bars, 100 μm.

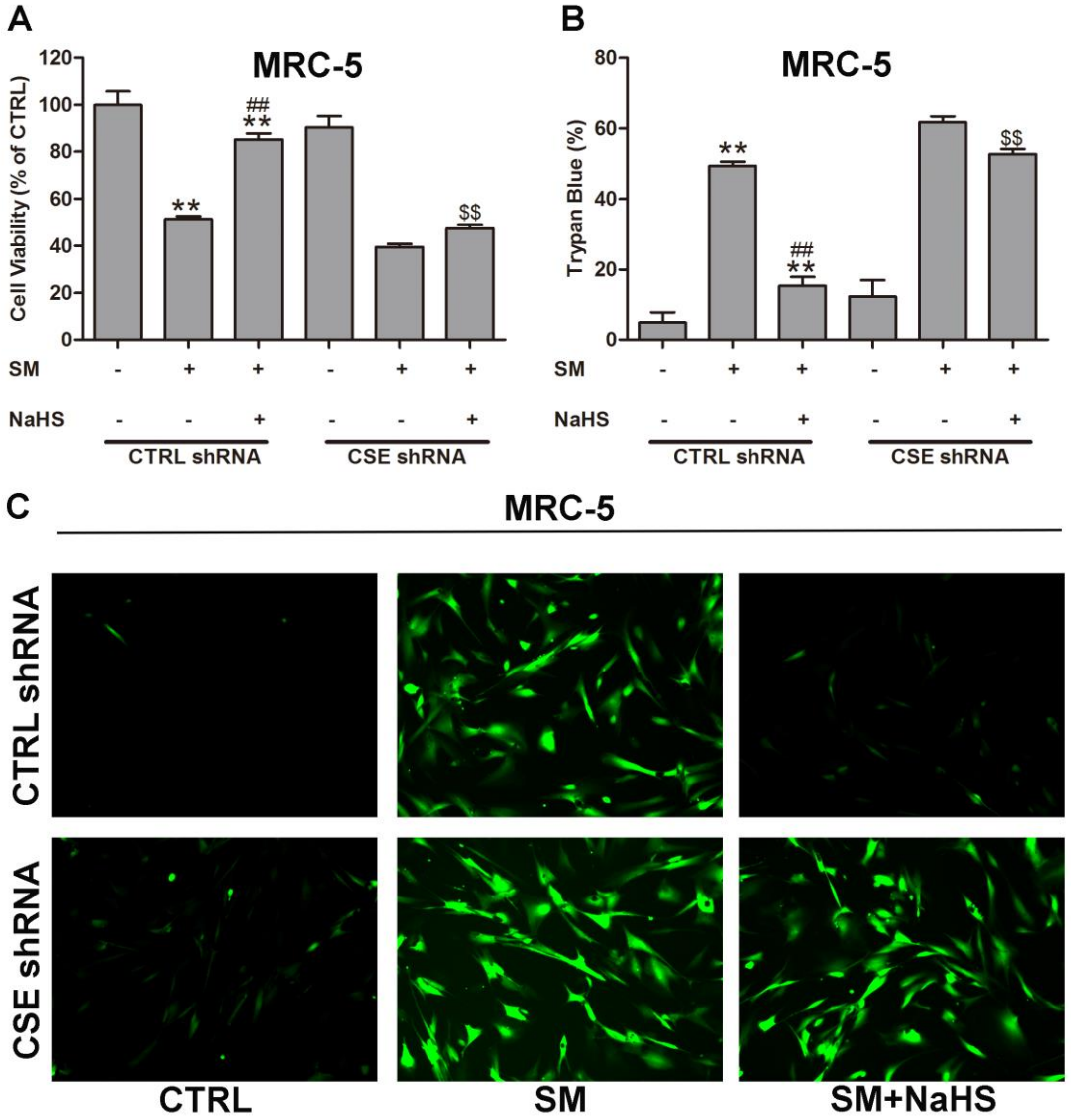

***Supplementary* Fig. 2.** Both wild type and CSE knockdown cells were treated with SM (50 μM), with or without NaHS (70 μM), and cell viability was analyzed by the CCK-8 assay (A). Cell death was detected by trypan blue staining (B). ROS production was detected by DCFH-DA fluorescence measurement (C). Experiments were repeated three times to ensure consistency of the results. Data are presented as the mean ± SEM (n=5). $^{**}p<0.01$ vs CTRL shRNA; $^{\#\#}p<0.01$ vs CTRL shRNA with SM; $^{\$\$}p<0.01$ vs CTRL shRNA with SM plus NaHS.

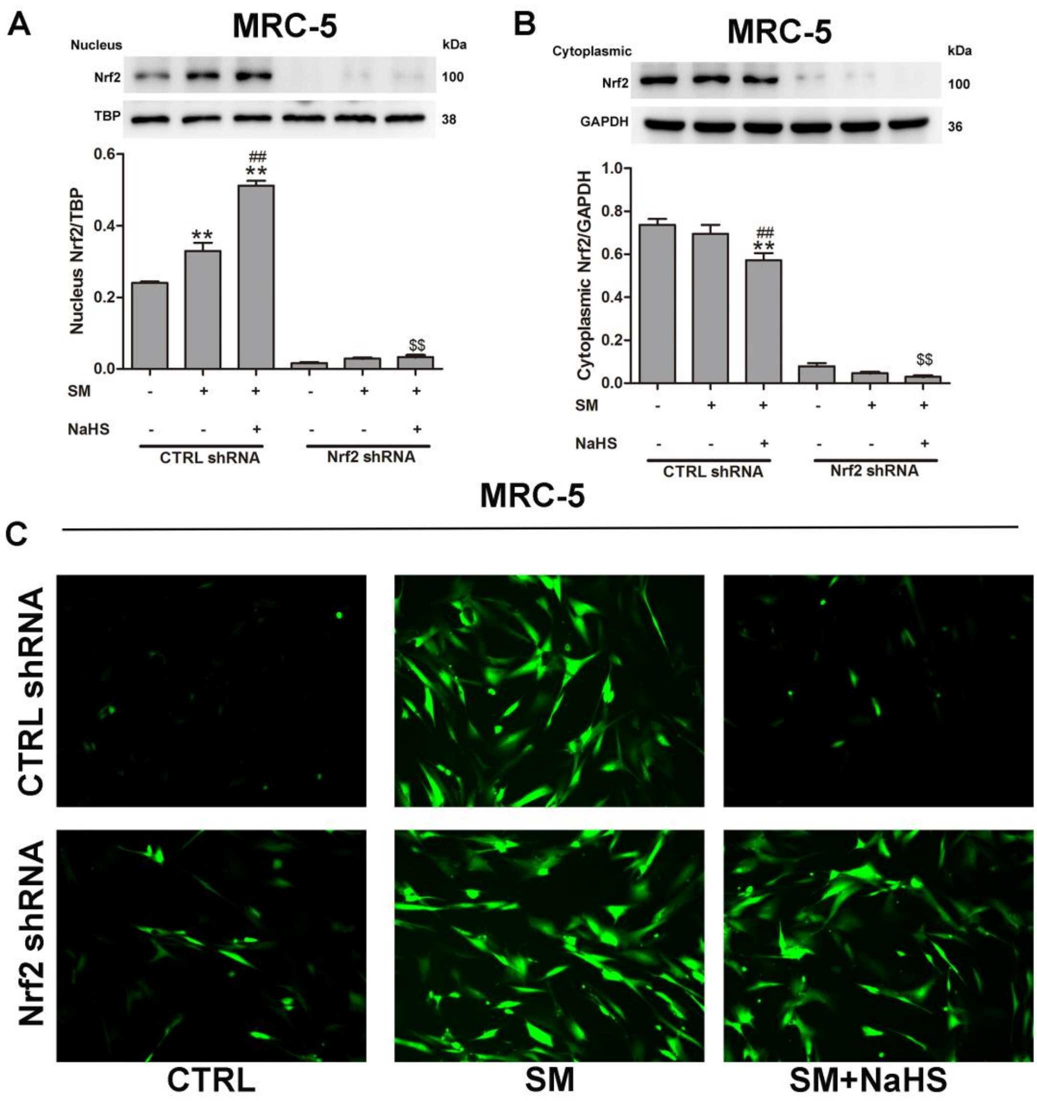

***Supplementary* Fig. 3.** MRC-5 cells were transfected with control shRNA or Nrf2 shRNA for 48 h and then treated with SM (100 μM) in the presence or absence of NaHS (70 μM) for 24 h. Cells were collected for isolation of nuclear and cytosolic proteins. Nrf2 protein levels in cells were determined by western blot analysis (A, B). ROS production was detected by DCFH-DA fluorescence measurement (C). Data are presented as the mean ± SEM(n=5). $^{**}p<0.01$ vs CTRL shRNA; $^{\#\#}p<0.01$ vs CTRL shRNA with SM; $^{\$\$}p<0.01$ vs CTRL shRNA with SM plus NaHS.

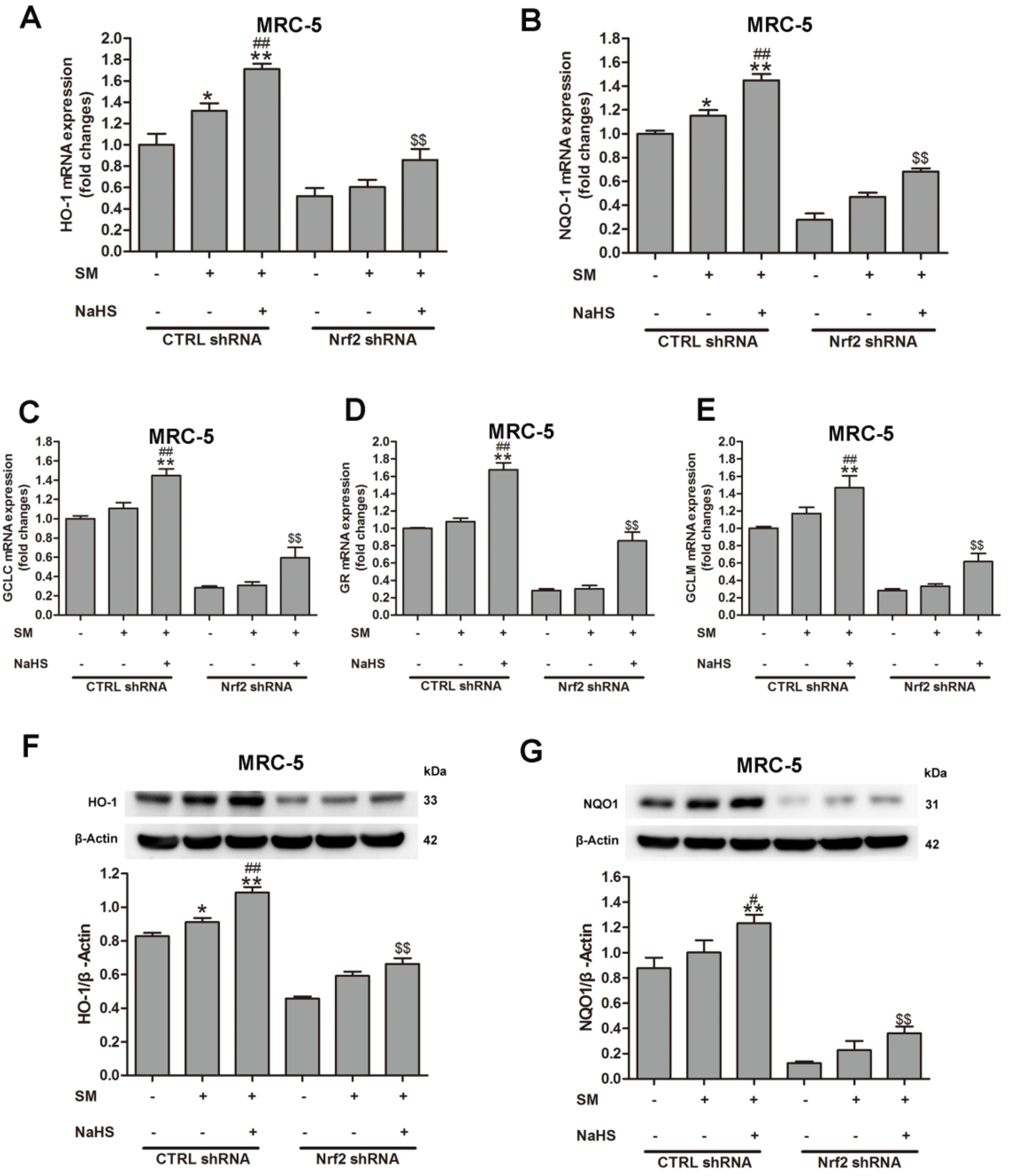

Supplementary **Fig. 4.** MRC-5 cells were transfected with control shRNA or Nrf2 shRNA for 48 h and then treated with SM (100 μM) in the presence or absence of NaHS (70 μM) for 24 h. The mRNA levels of Nrf2 downstream target cytoprotective genes including HO-1 (A), NQO1 (B), GCLC (C), GR (D), and GCLM (E) in cells were determined by RT-PCR. The protein levels of Nrf2 downstream, including HO-1 (F) and NQO1 (G) in cells were determined by western blot. Data are presented as the mean ± SEM(n=5). *p<0.05 vs CTRL shRNA; **p<0.01 vs CTRL shRNA; $^{\#}$p<0.05 vs CTRL shRNA with SM; $^{\#\#}$p<0.01 vs CTRL shRNA with SM; $^{\$\$}$p<0.01 vs CTRL shRNA with

SM plus NaHS.

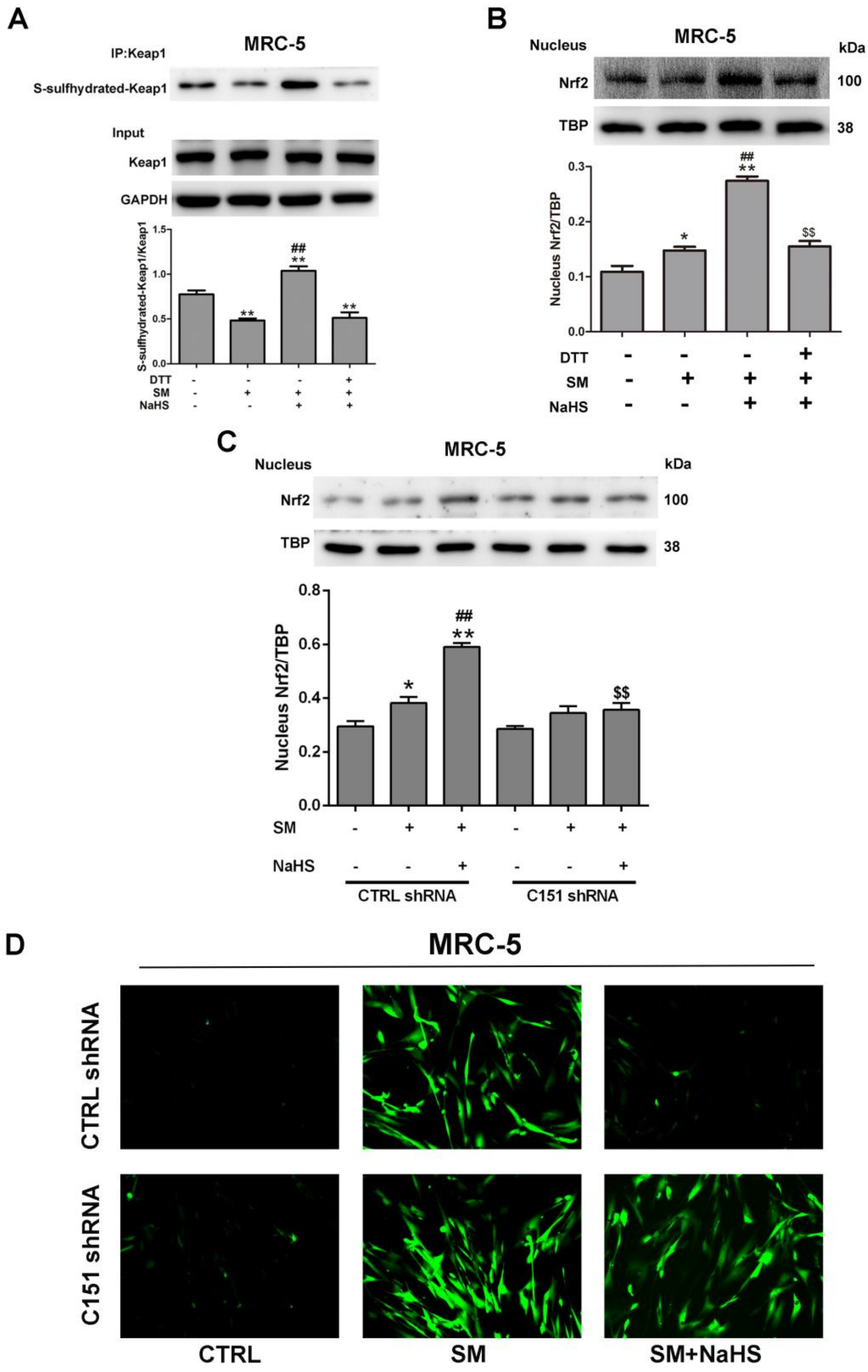

Supplementary **Fig. 5.** Cells were treated with SM (100 μM), with or without NaHS (70 μM) or DTT (1 mM) pretreatment. S-sulfhydrylation of Keap1 was detected using the "Tag-Switch"

method (A). Cells were collected for isolation of nuclear proteins. Nrf2 protein levels in cells were determined by western blot analysis (B, C). ROS production was detected by fluorescence measurement of the reported DCFH-DA (D). Experiments were repeated three times to insure consistency of results. Data are presented as the mean ± SEM(n=5). *$p<0.05$ vs untreated control; **$p<0.01$ vs untreated control; $^{##}p<0.01$ vs treatment with SM; $^{\$\$}p<0.01$ vs treatment with SM plus NaHS.
